# Limited nasal IFN production contributes to delayed respiratory virus clearance and suboptimal vaccine responses

**DOI:** 10.1172/jci.insight.182836

**Published:** 2025-09-16

**Authors:** Jorna Sojati, Olivia B. Parks, Taylor Eddens, Jie Lan, Monika Johnson, John V. Williams

**Affiliations:** 1 Department of Pediatrics and; 2 Department of Microbiology & Molecular Genetics, University of Pittsburgh School of Medicine, Pittsburgh, Pennsylvania, USA.; 3 Institute for Infection, Immunity, and Inflammation in Children, UPMC Children’s Hospital of Pittsburgh, Pittsburgh, Pennsylvania, USA.; 4 University of Wisconsin–Madison School of Medicine and Public Health, Madison, Wisconsin, USA.

**Keywords:** Immunology, Infectious disease, Virology, Influenza, Innate immunity, Vaccines

## Abstract

Acute lower respiratory infections are the primary cause of global mortality in postneonatal children. Most respiratory viruses primarily involve upper airway infection and inflammation, yet nasal responses are poorly characterized. Using a mouse model of human metapneumovirus (HMPV), we found viral burden was higher in nasal airways and exhibited delayed clearance. Despite high burden, there was low nasal expression of type I and III interferon (IFN). Single-cell RNA-sequencing (scRNA-Seq) from HMPV-infected mice showed lower nasal IFN-stimulated gene (ISG) expression and nasal enrichment of genes negatively regulating IFN. scRNA-Seq of patients with COVID-19 verified lower ISG expression in upper airways. HMPV infection downregulated nasal expression of IFN regulatory factor 3, suggesting a mechanism for limited response. To rescue the quiescent environment, we administered type I or III IFN to upper airways early postinfection, leading to lower nasal HMPV titer and virus-specific CD8^+^ T cell upregulation. Intranasal immunization adjuvanted with type I or III IFN improved immune response, reduced clinical disease, and enhanced viral clearance in HMPV and influenza infection. IFN adjuvant increased recruitment of dendritic cells, recruitment of resident memory T cells, and neutralizing antibodies. These findings reveal locally suppressed IFN production contributes to a quiescent nasal immune landscape that delays viral clearance and impairs mucosal vaccine responses.

## Abstract 2

We found that despite high metapneumovirus titer in the nasal tissue, interferon responses were limited, which likely impairs the response to mucosal vaccination against respiratory viruses.

## Introduction

Acute respiratory infections (ARIs) are a leading cause of hospitalization and death in children worldwide, accounting globally for 15% of all deaths in children under 5 years old (1–3). Human metapneumovirus (HMPV) is a primary cause of upper and lower airway infection in children, contributing to 4%–16% of ARI worldwide (4–6). Although nearly all people are infected with HMPV by 5 years old, reinfections occur often, highlighting difficulty in developing long-lasting immunity (5, 7). HMPV also contributes substantially to morbidity and mortality in adults, particularly immunocompromised persons and adults older than 65 (8, 9). HMPV commonly presents as mild, self-limiting upper airway illness but also causes more severe lower airway disease leading to hospitalization, such as bronchiolitis and pneumonia (5, 10–13).

We and others have shown that type I and III interferon (IFN) are critical to drive HMPV pathology and restrict lung viral replication (14–17). IFNs are essential to prime antiviral T cell responses (16, 18, 19), and both lung CD8^+^ and CD4^+^ T cells contribute to HMPV clearance (20). HMPV promotes recruitment of virus-specific CD8^+^ T cells in the lung, though these exhibit programmed cell death 1–mediated (PD-1–mediated) functional impairment (21–23). However, HMPV and other respiratory viruses, including influenza, rhinovirus, respiratory syncytial virus (RSV), and SARS-CoV-2 most often cause self-limited disease restricted to the upper tract (24–26). Little is known about IFN or CD8^+^ T cell responses to HMPV in the upper airway. Since studies of human immune responses to respiratory viruses are most feasible through nasal or nasopharyngeal sampling, this leaves a translation gap between the laboratory and the clinic (12, 27–29).

Many respiratory viruses infect nasal epithelial cells (30–34), and IFNs are produced in nasal airways in response to respiratory viral infection (35–38). However, there are different correlations between IFN and disease severity; higher nasal type I IFN correlated with milder disease for RSV and SARS-CoV-2, while influenza virus showed correlation of nasal type I IFN levels with more severe disease (35, 36, 38). The local immune response is also important for mucosal intranasal vaccines; these offer a promising noninvasive route of immunization for respiratory viruses, but existing nasal vaccines show limited therapeutic efficacy (39, 40).

Here, we discovered a quiescent nasal immune environment characterized by low IFN expression and limited antiviral IFN responses despite high viral burden. We show that nasal airways of HMPV-infected mice are enriched for genes that suppress IFN production and found that nasal IFN regulatory factor 3 (IRF3) expression was downregulated by HMPV infection. This quiescent nasal landscape could be rescued and viral clearance enhanced by intranasal delivery of exogenous IFN. Nasal immunization also required adjuvant with type I or III IFN to promote CD8^+^ T cell memory formation, elicit potent neutralizing antibodies, and reduce clinical disease. These data uncover limited (and potentially suppressed) IFN production as a key contributor to nasal immune quiescence and highlight IFNs as promising adjuvants for intranasal mucosal vaccines.

## Results

### Nasal airways show delayed viral clearance, minimal IFN production, and limited immune response to infection.

**Figure 1:**
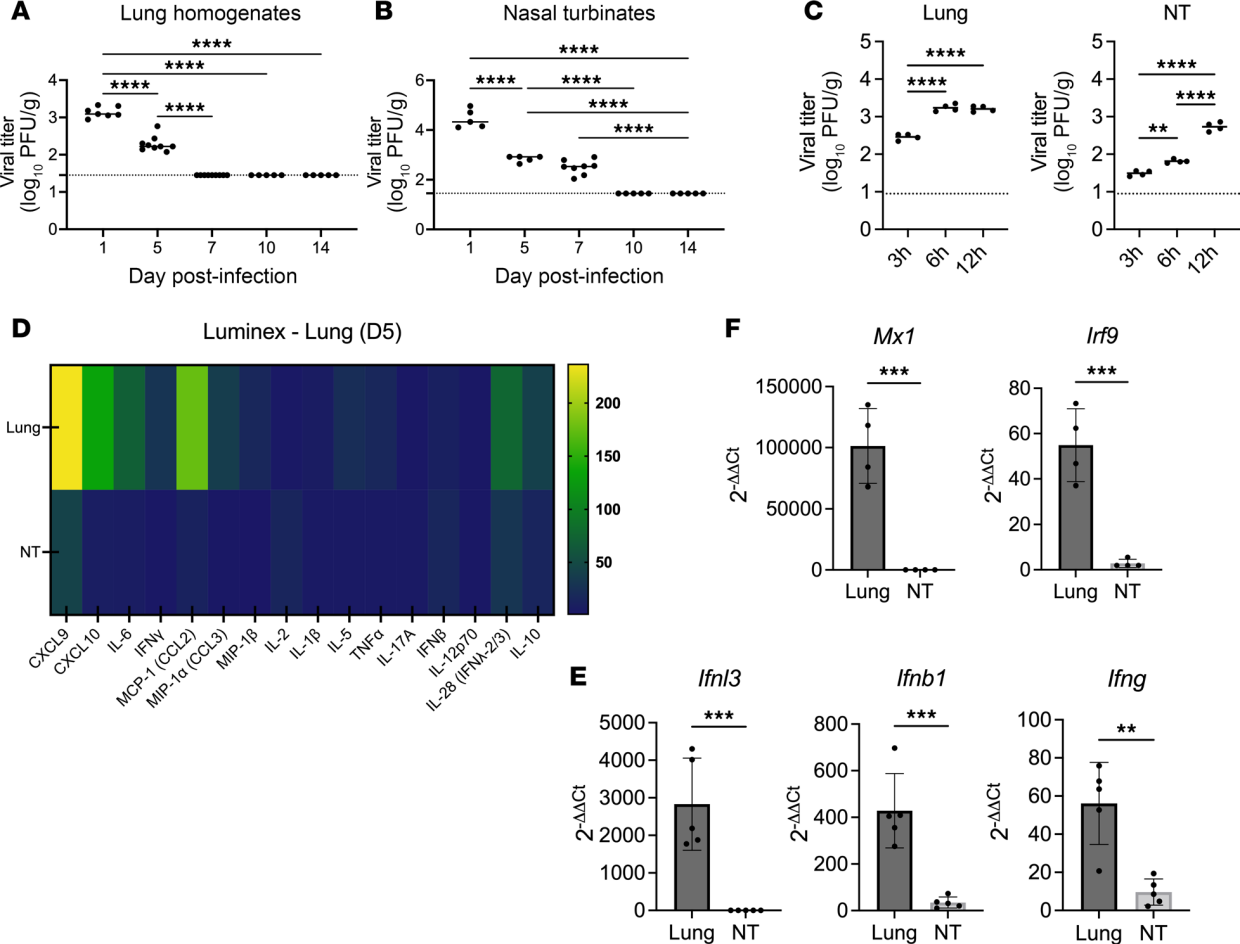
Upper airways show delayed HMPV clearance, minimal IFN production, and a quiescent immune profile. Mice were infected with 5 × 10^5^ PFU C2-202 HMPV. HMPV titer (PFU/g) was measured in lung homogenates (**A**) or nasal turbinates (**B**) of HMPV-infected mice out to day 14 postinfection. (**C**) HMPV titer (PFU/g) was also measured in lung homogenates (left) or nasal turbinates (right) at early time points of 3, 6, and 12 hours postinfection. Limit of detection noted by dashed black line. Analyses for **A**–**C** done by 1-way ANOVA. (**D**) Protein expression levels (ng/mL) of inflammatory cytokines in lung or nasal turbinate homogenates collected day 5 postinfection were assessed by Thermo Fisher Scientific Luminex immunoassay and represented by heatmap. (**E**) *Ifng*, *Ifnb1*, and *Ifnl3* and (**F**) *Irf9* and *Mx1* expression in lung and nasal turbinate homogenates of HMPV-infected mice was measured by qPCR day 1 postinfection. Data were normalized to the HPRT1 gene and mock-infected mice using the 2^–ΔΔCt^ method. Analyses done by Student’s *t* test. **P < 0.01, ***P < 0.001, ****P < 0.0001.

We first sought to establish the kinetics of HMPV replication in the upper airways using a well-established mouse model of acute upper and lower tract infection. We infected mice intratracheally with virulent strain C2-202 at an inoculum that causes clinical disease (~10% weight loss) and resolves within 10–14 days, similar to human infection (14, 15, 23). High HMPV titers in both upper and lower airways were detected early postinfection (Figure 1, A and B). However, while HMPV was cleared from the lungs by day 7, virus was not cleared from the nose until day 10 postinfection (Figure 1B). We also examined HMPV replication kinetics at several earlier time points in upper and lower airways, showing that lung HMPV titers peak by 6 hours postinfection, while nasal airways show increasing titers out to 12 hours postinfection (Figure 1C). We assessed inflammatory cytokine profiles in the upper and lower airways of HMPV-infected mice by Luminex immunoassay. While lower airways showed upregulation of several inflammatory cytokines with HMPV infection, upper airways maintained a minimal cytokine profile despite high HMPV burden (Figure 1D). We then sought to assess whether this observed quiescent nasal landscape correlated with expression of IFN. We measured levels of type I (IFN-β), type II (IFN-γ), and type III (IFN-λ) IFN transcripts by quantitative PCR (qPCR), observing high upregulation of type I and III IFN and mild upregulation of type II IFN in mouse lungs by day 1 postinfection (Figure 1E). In contrast, nasal IFN upregulation was not observed and IFN transcripts were minimally detectable in nasal turbinates (Figure 1E). We also measured nasal IFNs at several time points postinfection by Luminex assay and observed little or undetectable levels of IFN-β, IFN-γ, or IFN-λ to day 7, suggesting this was not limited to day 1 postinfection (Supplemental Figure 1; supplemental material available online with this article; https://doi.org/10.1172/jci.insight.182836DS1). To verify the downstream signaling effects, we measured ISG and found lung upregulation of IFN-stimulated genes (ISGs) MX1 and IRF9 but little ISG expression in the nose (Figure 1F). Taken together, these data show that in contrast with lower airways, the upper respiratory tract showed minimal inflammatory cytokine and IFN expression despite high HMPV burden.

We next characterized innate and adaptive immune subsets in the upper airway using flow cytometry. We developed a new protocol for obtaining nasal immune cells from processing of turbinates for flow cytometric analysis of myeloid cells and lymphocytes (Supplemental Figure 2, A and B). In contrast with prior data showing an increase in lung DCs, macrophages, and monocytes on day 1 postinfection, we found here that, despite the presence of basal nasal myeloid cells, there was no upregulation of nasal DCs, macrophages, or monocytes with HMPV (Supplemental Figure 3, A–G). Similarly, we did not observe increase of nasal lymphocyte subsets, including B cells, NK cells, and CD4^+^ and CD8^+^ T cells, by day 10 postinfection (Supplemental Figure 3, H–L). Collectively, these data indicate a quiescent nasal environment with low IFN and inflammatory cytokine expression and minimal immune cell recruitment in HMPV infection.

### Human and mouse sequencing data verify low IFN response in upper airway.

**Figure 2:**
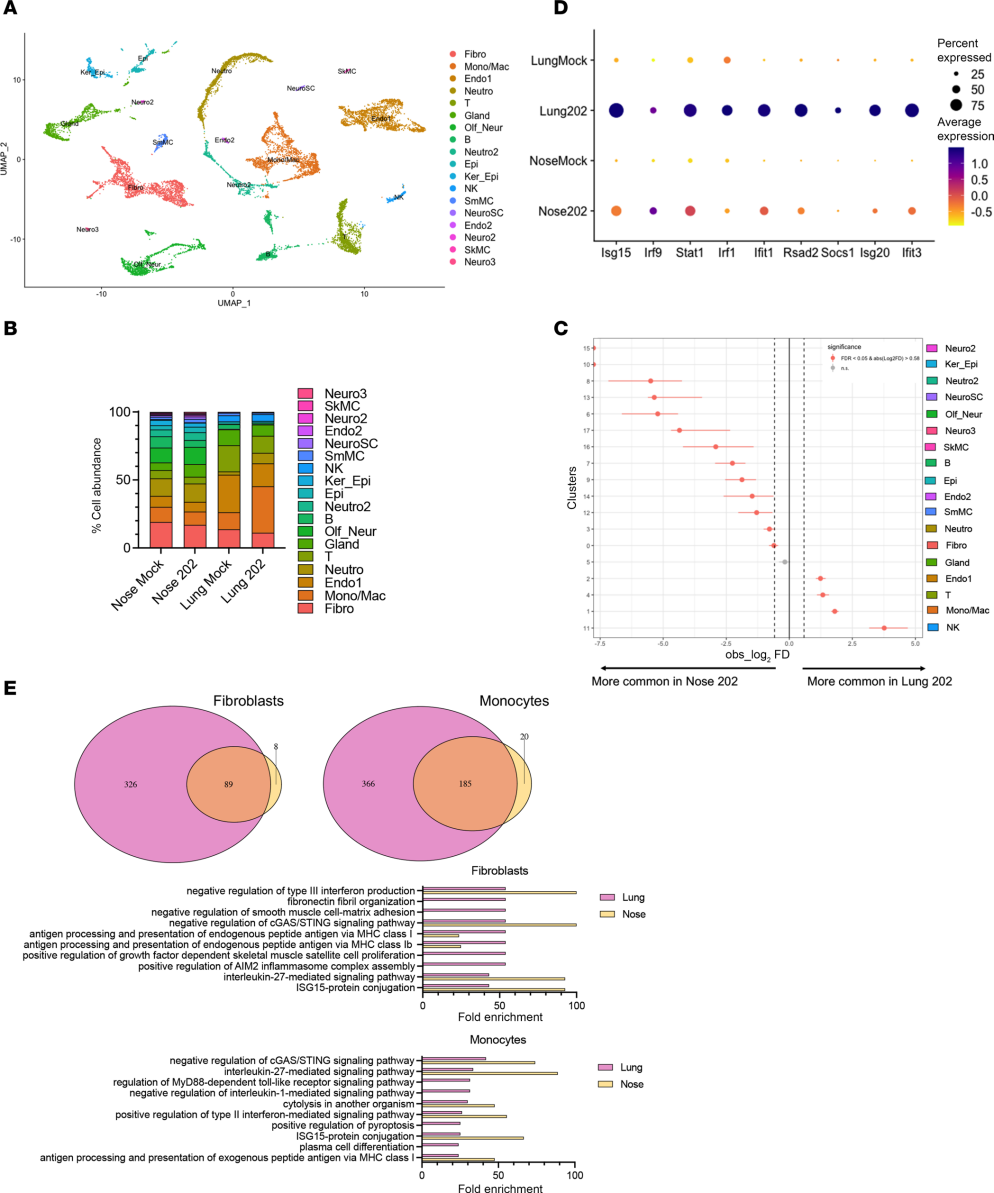
Upper airways of HMPV-infected mice are enriched for genes negatively regulating IFN expression. Single-cell RNA sequencing (scRNA-Seq) was performed on cells isolated from nasal turbinates or lungs of mice infected with 5 × 10^5^ PFU C2-202 HMPV (or mock-infected with LLC-MK2 cell lysate) and harvested day 1 postinfection. Two mice per group (2 mock-infected, 2 HMPV-infected) were used for the scRNA-Seq experiment. (**A**) A total of 18 distinct subpopulations of immune and epithelial cells were defined and represented by Uniform manifold approximation and projection (UMAP). (**B**) Differential abundance of immune cell populations shown for lung and nasal samples from mock- or HMPV-infected mice. (**C**) Relative abundance levels for lung versus nasal samples from HMPV-infected mice. (**D**) Expression of several ISGs shown by dot plots for lung and nasal samples from mock- or HMPV-infected mice. (**E**) Gene Ontology enrichment analysis was done for 2 predominant immune cell subpopulations in both lung and nasal samples, and top 10 differentially expressed genes are shown. Fibroblasts and monocytes in nasal airway showed enrichment for pathways that negatively regulate IFN production. SkMC, skeletal muscle cells; SmMC, smooth muscle cells.

To further compare the immune landscape of the upper and lower airway, we performed single-cell RNA sequencing (scRNA-Seq) of lung and nasal cells from HMPV-infected mice day 1 postinfection. We observed the presence of 18 cell populations combined (Figure 2A and Supplemental Figure 4A). While some populations were shared, there were noticeable differences in cell subsets and abundance between upper and lower airway. Many cell populations, including fibroblasts, monocytes, B and T lymphocytes, NK cells, and neutrophils, were present in both the lung and nose, while some neuronal and neuroendocrine populations (likely from olfactory mucosa) were found only in nasal samples (Figure 2B and Supplemental Figure 4, A and B). HMPV-infected mouse lungs were enriched in NK cells, macrophages, monocytes, and T cells, while HMPV-infected mouse noses were enriched in nasal specific neuronal, epithelial, and B cells (Figure 2C). Furthermore, while HMPV infection led to a robust increase in monocyte, macrophage, and neutrophil populations in lungs, this was not observed in the nose (Figure 2, B and C, and Supplemental Figure 4C). We saw modest HMPV-induced increases in expression of several ISGs in mouse lung but not nasal airway with HMPV infection (Figure 2D). Gene Ontology analysis showed fewer genes enriched in upper airway with HMPV infection (Figure 2E). However, some of the most highly enriched genes in nasal airway function to negatively regulate type III IFN and the cyclic GMP-AMP synthase (cGAS)/stimulator of interferon genes (STING) pathway, both critical for promoting IFN responses (Figure 2E). Thus, nasal airway failed to recruit myeloid cells, showed low ISG expression, and enriched genes that negatively regulate innate immune responses.

**Figure 3:**
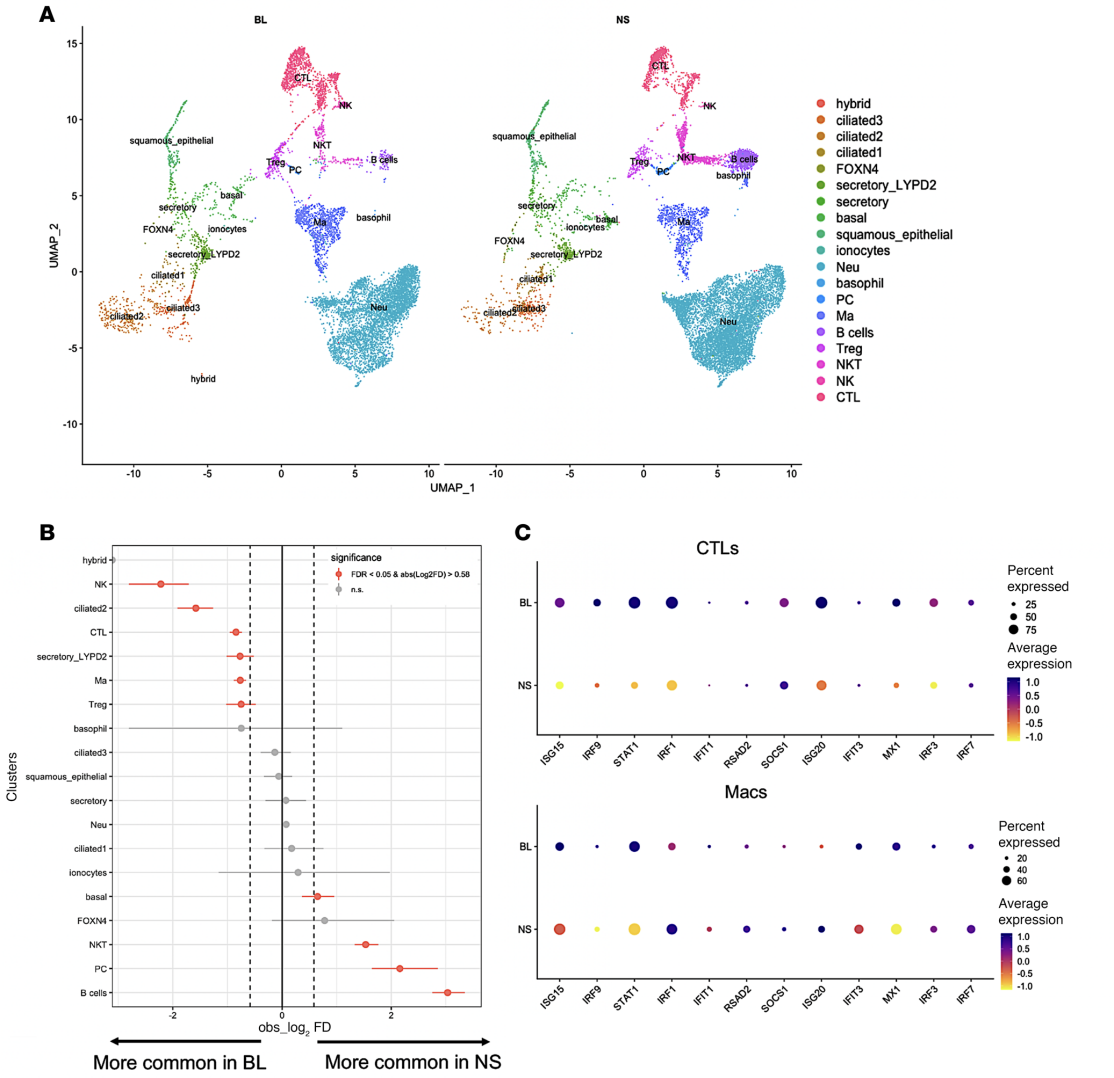
Upper airways of patients with severe COVID-19 show reduced IFN responses. We analyzed an existing scRNA-Seq dataset of samples collected from the lower airway (bronchoalveolar lavage; BL) and upper airway (nasal swab; NS) of the same patient for 2 patients with severe COVID-19. (**A**) A total of 19 distinct subpopulations of immune and epithelial cells were defined, represented by UMAP. (**B**) Differential abundance of immune cell populations for BL and NS from patients with COVID-19. (**C**) Expression of several ISGs in cytotoxic T lymphocytes (CTLs) and macrophages shown by dot plots for BL and NS of patients with COVID-19. PC, plasma cell; Ma, macrophage.

To test whether these findings were observed with human respiratory virus infection, we analyzed an existing scRNA-Seq dataset of bronchoalveolar lavage (BAL) and nasal swab (NS) samples collected from the same patients with severe COVID-19 infection (41). Similar to the mouse data, many immune cell populations were present in both upper and lower airway but with distinct differences in cell abundance (Figure 3A). Lower airways of patients with COVID-19 had increased abundance of macrophages, CTLs (CD8^+^ T cells), and NK cells compared with upper airway, which exhibited smaller but detectable populations of these cell types (Figure 3B). Similar to the analysis previously performed, we assessed CTLs and macrophages for changes related to ISGs. CTLs and macrophages from BAL showed higher ISG expression than cells from NS, suggesting greater IFN responses in the lower airways of patients with COVID-19 (Figure 3C).

Taken together, these mouse and human sequencing data provide support for the presence of a quiescent nasal immune environment and suggest that this quiescent landscape may affect immune responses to other respiratory viruses. Furthermore, these data suggest that the observed quiescent nasal immune environment is modulated, in part, by a diminished IFN response, with mouse scRNA-Seq highlighting nasal enrichment of genes that negatively regulate IFN production.

### IRF3 production is suppressed during HMPV infection in nasal airways.

**Figure 4:**
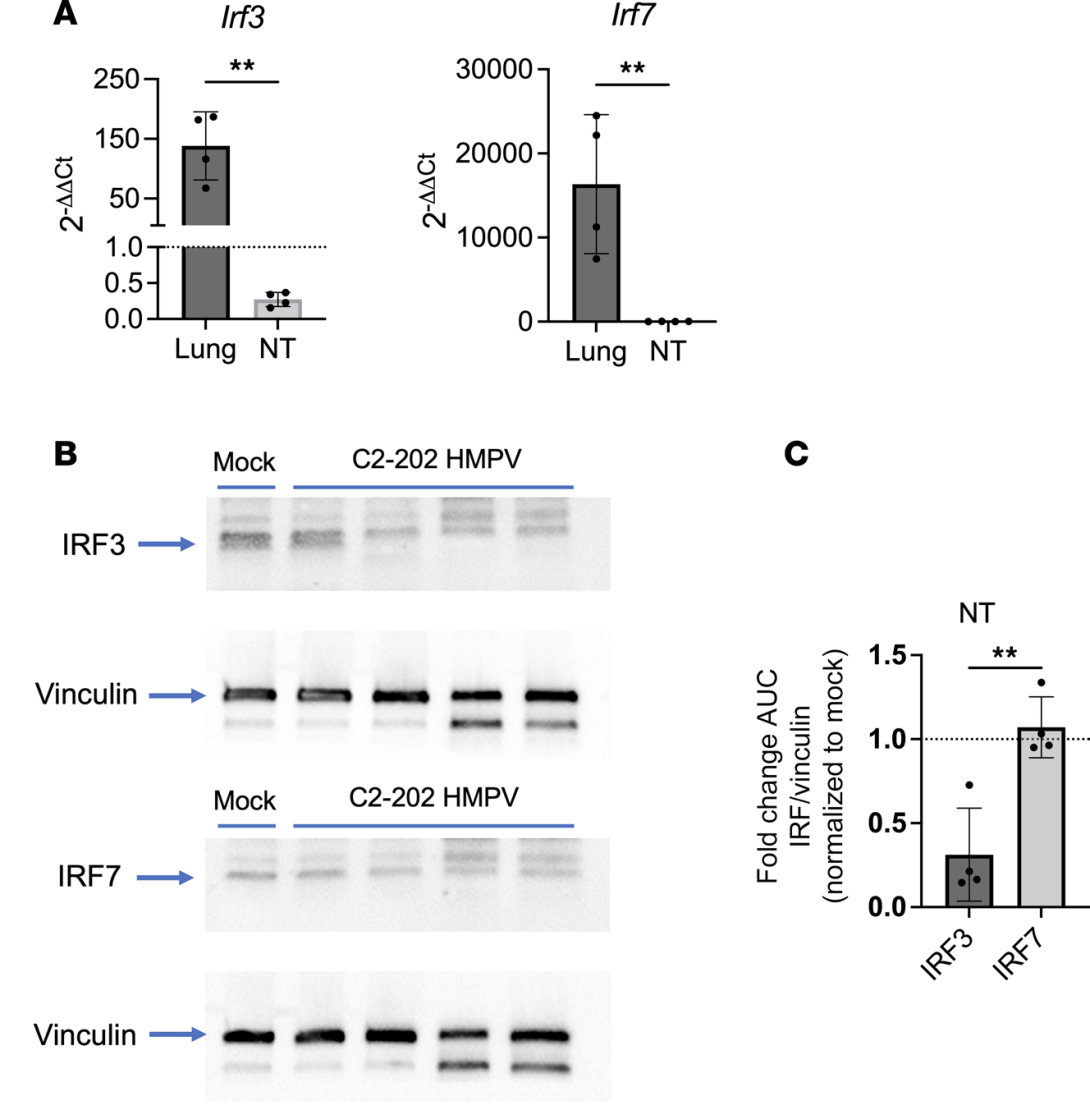
HMPV-infected upper airways suppress IFN production through downregulation of IRF3. Mice were infected with 5 × 10^5^ PFU C2-202 HMPV, and expression of *Irf3* and *Irf7* was assessed day 1 postinfection in lungs and nasal turbinates by qPCR (**A**). Data were normalized to the HPRT1 gene and mock-infected mice by the 2^–ΔΔCt^ method. (**B**) IRF3 and IRF7 protein levels were measured in nasal turbinates of mice infected with HMPV or mock-infected by Western blot. Data were normalized to vinculin. (**C**) Fold-change in AUC measurements of normalized IRF3 and IRF7 expression in mock- versus HMPV-infected nasal turbinates. Samples from 4 HMPV-infected mice were used for this experiment. Analyses done by Student’s *t* test. **P < 0.01.

To understand the low nasal IFN production during HMPV infection, we measured transcripts of IRF3 and IRF7, transcription factors directly upstream of IFN production. Both IRF3 and IRF7 were upregulated in lungs, but not nose, with infection (Figure 4, A and B, and Supplemental Figure 5). In contrast, IRF7 showed no upregulation in nasal airways. Moreover, nasal IRF3 transcript levels were downregulated by HMPV infection (Figure 4A). We verified these findings in the nose and lungs by both qPCR and Western blot analysis, notably showing negative fold-change values for IRF3 expression in the nose and confirming suppressed nasal IRF3 production with HMPV infection (Figure 4C and Supplemental Figure 5). This provides a possible mechanism of suppressed IFN production, and subsequently minimal IFN response, in HMPV-infected nasal airways.

To further support a role for IRF3 in potential suppression of IFN, we performed siRNA-mediated knockdown studies of *Irf3* in C10 mouse alveolar type II cells, a well-validated in vitro model of HMPV infection (15). We first confirmed knockdown of Irf3 by qPCR, showing no expression of Irf3 in siRNA-treated C10 cells (Supplemental Figure 6A). Using this model, we measured levels of type I and III IFN by qPCR. While expression of type I (*Ifnb1*) and III (*Ifnl3*) IFN transcripts was increased in response to HMPV infection, levels of both IFNs were diminished in *Irf3*-siRNA knockdown cells infected with HMPV (Supplemental Figure 6, B and C). These data support a key role for IRF3 in promoting type I and III IFN production in response to HMPV infection.

Additional studies were done to assess the role of IRF3 downregulation and its impact on antiviral function. To better understand kinetics of the observed IRF3 downregulation in response to HMPV infection, we measured levels of IRF3 and IRF7 by qPCR at earlier time points postinfection. In the lungs, IRF3 and IRF7 expression were both increased by 12 hours postinfection (Supplemental Figure 7A). In the nasal airway, IRF7 expression remained unchanged, while IRF3 expression was initially upregulated at 3 hours postinfection but significantly decreased by 12 hours postinfection (Supplemental Figure 7B). While IRF3 (and IRF7) have well-known roles in upstream promotion of IFNs, we also tested whether IRF3 and IRF7 expression increased in response to direct IFN treatment. We treated uninfected mice with either type I or III IFN (delivered intranasally in 50 μL volume for whole airway administration), and measured levels of IRF3 and IRF7 by qPCR. Notably, both IRF3 and IRF7 levels were increased by type I or III IFN treatment (Supplemental Figure 7C).

These data highlight a possible mechanism of IFN downregulation through IRF3 suppression in response to HMPV infection and highlight a critical role for IRF3 in antiviral immunity by promoting expression of type I and III IFNs.

### Priming of nasal airways with type I or III IFN enhances antiviral immunity.

**Figure 5:**
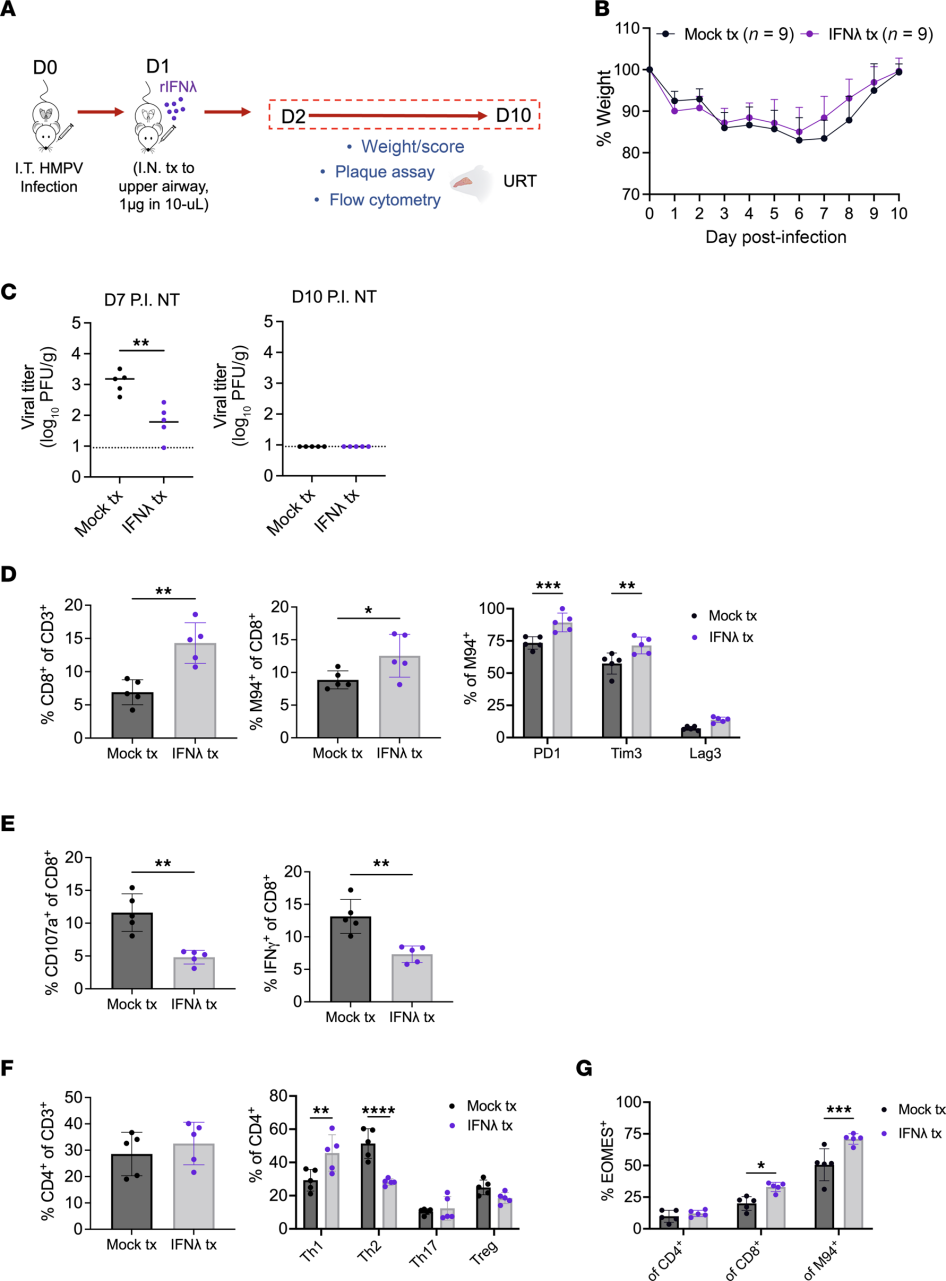
Type III IFN treatment of upper airway increases HMPV-specific CD8^+^ T cell recruitment and enhances viral clearance. Mice were first infected with 5 × 10^5^ PFU C2-202 HMPV intratracheally, followed by an intranasal treatment with recombinant mouse IFN-λ (1 μg in 10 μL) on day 1 postinfection (or mock treatment with same volume 0.1% BSA) as shown by schematic (**A**). Disease was assessed by measuring weight (**B**) to day 10 postinfection, represented as % of day 0. HMPV titer (PFU/g) was measured in nasal turbinates of mock- or IFN-λ–treated mice on day 7 (**C**) and 10 (**D**) postinfection. Limit of detection noted by dashed black line. (**D**–**G**) HMPV-infected mice receiving either mock or IFN-λ treatment were euthanized day 7 postinfection, and immune responses were assessed. (**D**) Frequency of total nasal (left), virus-specific (M94^+^) (middle), and inhibitory receptor–expressing HMPV-specific CD8^+^ T cells (right). (**E**) Frequency of nasal CD8^+^ T cells expressing CD107a (left) or IFN-γ (right) after 5-hour ex vivo stimulation with HMPV M94 peptide. (**F**) Frequency of total nasal CD4^+^ T cells (left) and CD4^+^ subsets (right) Th1 (% Tbet^+^ of CD4), Th2 (% GATA3^+^ of CD4), Th17 (% RORγT^+^ of CD4), and Treg (% FoxP3^+^ of CD4). (**G**) Frequency of EOMES expression (of total CD4, of total CD8, and of total M94-specific CD8). For this experiment 5 mock-treated and 5 mouse IFN-λ-treated mice were used. Analyses by Student’s *t* test (**C**–**E**) or 2-way ANOVA (**F** and **G**). *P < 0.05, **P < 0.01, ***P < 0.001, ****P < 0.0001. Tim3, T cell immunoglobulin mucin receptor 3; Lag3, lymphocyte activation gene 3.

We next tested whether we could improve nasal immune responses by priming with recombinant IFN. We previously showed that type I and III IFN in the lower airways drastically increase by day 1 postinfection and diminish afterward (15). We sought to mimic lung dynamics of IFN upregulation by delivering IFN in an upper tract–restricted fashion day 1 postinfection (15). We adapted this method to deliver recombinant IFN, optimizing delivery dose based on nasal ISG induction (Supplemental Figure 8). We first tested IFN-λ treatment, as IFN-λ is shown to cause fewer side effects than type I IFN (Figure 5A) (15, 42, 43). Although there was no reduction of clinical disease, nasal HMPV burden was significantly reduced with IFN-λ treatment (Figure 5, B and C). Additionally, IFN-λ treatment led to an increase in both total and virus-specific nasal CD8^+^ T cells on day 7 postinfection (Figure 5D). Day 7 was chosen as a time point that highlights activation of adaptive immune responses, as evidenced by rapid viral clearance from the lungs because of T and B cell–driven antiviral immunity (Figure 1A). In HMPV-specific CD8^+^ T cells from IFN-λ–treated mice, there was increased expression of PD-1 and Tim3 (Figure 5D), as well as decreased degranulation and IFN-γ in CD8^+^ T cells stimulated with HMPV peptide (Figure 5E). These findings suggest IFN-λ treatment promoted CD8^+^ T cell recruitment and activation, but also induced acute CD8^+^ T cell impairment, as we previously described (21). While there was no increase in total CD4^+^ T cells, IFN-λ treatment promoted skewing of nasal CD4^+^ populations to a pro-inflammatory Th1 phenotype (Figure 5F). Eomesodermin (EOMES), a marker of CD8^+^ T cell terminal differentiation and exhaustion, was upregulated in IFN-λ–treated total and HMPV-specific CD8^+^ T cells (Figure 5G). Collectively, priming of the nasal airways with IFN-λ treatment resulted in enhanced viral clearance, recruitment and activation (along with subsequent acute impairment) of virus-specific CD8^+^ T cells, and skewing of CD4^+^ to pro-inflammatory Th1 T cells, suggesting a more efficient antiviral response. Similar findings were observed by priming the upper airway with type I IFN (IFN-β) treatment (Supplemental Figure 9), showing that both type I and III IFN can promote upper airway immunity. Furthermore, these phenotypes could also be observed with nasal type I or III IFN treatment at a later time point of day 10 postinfection (Supplemental Figure 10). Day 10 was chosen as a time point that represents rapid clearance of HMPV in nasal airways and presumed activation of adaptive immunity in the upper tract. Thus, delivery of type I or III IFN to the upper tract enhanced viral clearance, drove adaptive antiviral responses, and rescued the quiescent immune phenotype of nasal airway.

### IFN augmentation bolsters mucosal vaccine efficacy through innate and adaptive immune mechanisms.

**Figure 6:**
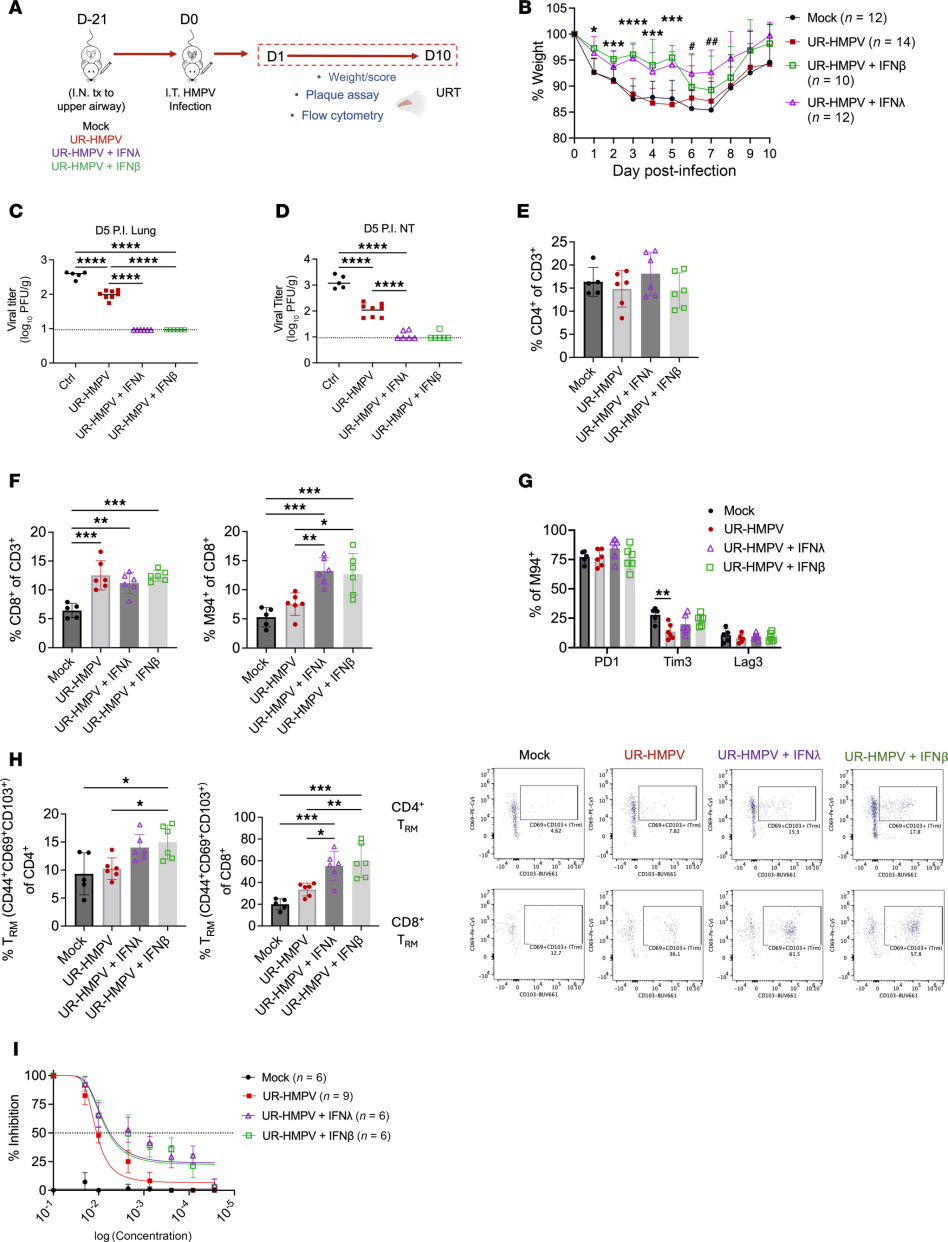
IFN adjuvant of nasal HMPV immunization enhances memory T cell responses and neutralizing antibody capacity to reduce clinical disease and viral burden. (**A**) Schematic of nasal immunization models. Mice were intranasally immunized with low-dose C2-202 HMPV (UR-HMPV), low-dose HMPV + adjuvant with IFN-λ (0.5 μg), low-dose HMPV + adjuvant with IFN-β (0.5 μg), or mock-immunized with LLC-MK2 cell lysate. Mice were challenged with HMPV infection 21 days postinoculation, and nasal responses to primary challenge were assessed. (**B**) Disease was assessed by measuring body weight to day 10 postinfection, represented as % of day 0. *P < 0.05, ***P < 0.001, ****P < 0.0001 for IFN-β– and IFN-λ–adjuvanted groups versus low-dose HMPV alone (UR-HMPV). ^#^*P* < 0.05, ^##^*P* < 0.01 for IFN-λ–adjuvanted group versus mock immunization. HMPV titer (PFU/g) was measured in lungs (**C**) and nasal turbinates (**D**) of immunized mice day 5 postinfection. Limit of detection noted by dashed black line. (**E**–**H**) Immunized mice were euthanized day 10 postinfection and immune responses assessed. For this experiment 5 mock-immunized, 6 HMPV-immunized, 6 IFN-λ–adjuvanted, and 6 IFN-β–adjuvanted mice were used. (**E**) Frequency of total nasal CD4^+^ T cells, (**F**) total nasal CD8^+^ T cells (left) and virus-specific (M94^+^) CD8^+^ T cells (right). (**G**) Frequency of inhibitory receptor–expressing M94^+^CD8^+^ T cells. (**H**) Frequency of nasal CD4^+^ and CD8^+^ T_RM_ cells (left) with representative flow cytometry plots (right). (**I**) Serum was collected from immunized mice day 5 postinfection, and neutralizing antibody responses were assessed by plaque reduction neutralization assay. Dashed black line denotes 50% inhibitory capacity (IC_50_). Analyses by 1-way or 2-way ANOVA. *P < 0.05, **P < 0.01, ***P < 0.001, ****P < 0.0001.

We next sought to determine what implications the observed immune quiescence of the upper airway had for mucosal vaccination, as well as whether IFN could boost vaccine efficacy. To do so, we developed a protocol for intranasal immunization by delivery of a low HMPV dose in an upper respiratory tract–restricted fashion 21 days prior to primary challenge as mock immunization, immunization with HMPV alone, or immunization with HMPV adjuvanted with type I (IFN-β) or III (IFN-λ) IFN (Figure 6A). All groups received primary challenge on day 0 with whole-airway HMPV infection as done for prior experiments. Only groups receiving HMPV immunization adjuvanted with IFN-λ and IFN-β showed less weight loss with primary challenge, suggesting IFN adjuvant was required to reduce clinical disease by vaccination (Figure 6B). While groups receiving HMPV immunization alone had lower lung and nose HMPV titers compared with mock vaccination, most mice that received HMPV adjuvanted with type I or III IFN showed no detectable HMPV burden in upper or lower airways by day 5 postinfection (Figure 6, C and D). No differences in CD4^+^ frequency were observed by immunization (Figure 6E). All groups receiving HMPV immunization showed an increase in total nasal CD8^+^ T cells by day 10 postinfection; however, groups adjuvanted with IFN showed higher levels of nasal virus-specific CD8^+^ T cells compared with HMPV vaccine alone (Figure 6F). However, these virus-specific cells did not exhibit increased levels of PD-1, Tim3, or Lag3 (Figure 6G). Mice receiving IFN-λ– and IFN-β–adjuvanted HMPV immunization demonstrated increases in both nasal CD8^+^ and CD4^+^ resident memory T cell (T_RM_) populations, showing greater memory responses with IFN adjuvant (Figure 6H). We also measured levels of serum neutralizing antibodies in different immunization groups. Mice receiving HMPV with IFN-λ or IFN-β adjuvant showed increased neutralizing antibody titer compared with HMPV vaccine alone (*P* = 0.0068 and *P* = 0.0092, respectively) (Figure 6I).

Given that HMPV clearance in both upper and lower airways was enhanced by intranasal immunization, we also assessed lung responses in mice immunized with HMPV (Supplemental Figure 11A). We did not observe increases in lung CD4^+^ or CD8^+^ T cells in immunized groups (Supplemental Figure 11, B and C). However, mice receiving IFN-λ– and IFN-β–adjuvanted immunization showed higher levels of lung HMPV-specific CD8^+^ T cells compared with HMPV alone (Supplemental Figure 11D). Furthermore, mice immunized with IFN-adjuvanted HMPV had increased lung CD4^+^ T_RM_, though levels of lung CD8^+^ T_RM_ did not increase with immunization (Supplemental Figure 11, E and F).

To employ a second and more clinically relevant model of HMPV vaccination, we delivered UV-inactivated HMPV (UV-HMPV) in a low dose using the same method of upper respiratory tract–restricted administration 21 days before primary challenge, creating groups of mock vaccination, vaccination with UV-HMPV alone, or vaccination with UV-HMPV adjuvanted with type I or III IFN. Similar to the first immunization model, we saw that only HMPV vaccine groups adjuvanted with type I or III IFN showed less weight loss with primary challenge, further supporting that type I and III IFN adjuvant is required to reduce clinical disease (Supplemental Figure 12A). All HMPV vaccine groups had decreased nasal titers, although notably a few mice in the IFN-adjuvanted groups showed no detectable HMPV titers in the upper airway by day 7 postinfection (Supplemental Figure 12B). Additionally, more mice in the IFN-adjuvanted group cleared HMPV in the lungs by day 7 postinfection, though this did not reach statistical significance (Supplemental Figure 12C). These data support a role for type I and III IFN adjuvant in reducing clinical disease and decreasing HMPV burden in upper and lower airways in an HMPV vaccine model.

We next sought to determine whether this decrease in clinical disease and viral burden was truly by adjuvant effects of IFN on HMPV immunization or was driven by IFN alone. We treated mice intranasally in an upper tract–restricted fashion with type I or III IFN alone 21 days prior to primary challenge. Mice receiving solely type I or III IFN intranasally before infection showed no changes in weight loss or in HMPV burden in upper and lower airways (Supplemental Figure 12, D–F). Thus, type I and III IFN act through an adjuvant effect on HMPV immunization to reduce clinical disease and HMPV burden.

**Figure 7:**
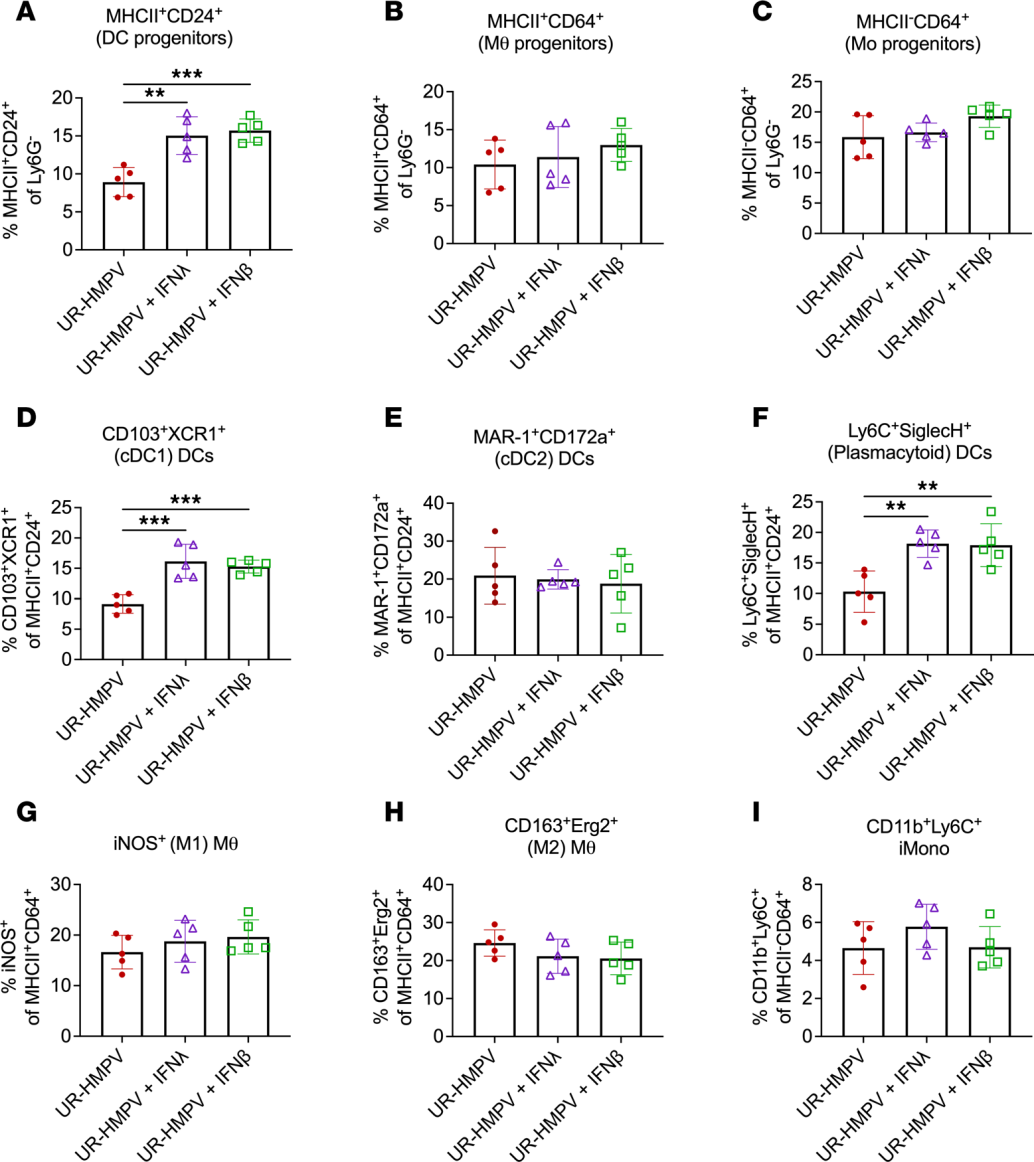
IFN adjuvant of nasal HMPV immunization increases nasal recruitment of cDC1 and pDC. Mice were immunized as described in Figure 6A with low-dose HMPV alone (UR-HMPV), HMPV adjuvanted with IFN-λ, or HMPV adjuvanted with IFN-β. 5 mice per group were used for this experiment. Mice were euthanized day 1 postimmunization, and nasal myeloid cell populations were quantified by flow cytometry. (**A**–**C**) Frequency of myeloid cell progenitors, including progenitors for DC (**A**), macrophage (**B**), and monocyte (**C**) lineages. (**D**–**F**) Frequency of DC subsets, including cDC1 (**D**), cDC2 (**E**), and pDC (**F**). (**G**–**I**) Frequency of macrophage and monocyte subsets, including M1 macrophages (**G**), M2 macrophages (**H**), and inflammatory monocytes (**I**). Analyses by 1-way ANOVA. **P < 0.01, ***P < 0.001. cDC1, conventional type I DC; pDC, plasmacytoid DC.

To uncover how IFN supplementation priming led to more efficient adaptive responses, we analyzed nasal myeloid cell subsets in groups receiving either upper tract–restricted HMPV alone or HMPV with IFN-λ or IFN-β adjuvant 1 day postimmunization. IFN-adjuvanted immunized groups showed upregulation of an MHCII^+^CD24^+^ DC progenitor population though not macrophage or monocyte precursors (Figure 7, A–C). Specifically, increases were seen in pro-inflammatory CD103^+^XCR1^+^ cDC1 and IFN-producing Ly6C^+^SiglecH^+^ pDC but not cDC2 (Figure 7, D–F). There were no differences in other macrophage and monocyte populations (Figure 7, G–I). Thus, IFN adjuvant primes adaptive immunity and enhances antiviral responses through upregulation of antigen-presenting cells, particularly cDC1 and pDC subsets.

To test whether these findings were specific to HMPV, we used a mouse whole-airway infection model with influenza virus A/PR/8/34 (PR8) (Supplemental Figure 13A). We observed high influenza virus burden in both upper and lower airway by day 5 postinfection (Supplemental Figure 13A). Similar to HMPV, there was mild weight loss (~10%) and clinical disease with PR8 (Supplemental Figure 13, B and C). Moreover, type I (IFN-β), type II (IFN-γ), and type III (IFN-λ) IFN transcripts were upregulated in the lung but not the nose, despite high nasal viral burden (Supplemental Figure 13, D–F).

We next adapted the intranasal IFN-adjuvanted immunization method to influenza. A low PR8 inoculum was delivered intranasally in an upper respiratory tract–restricted fashion 21 days prior to primary challenge as mock immunization, immunization with PR8 influenza virus alone, or immunization with PR8 adjuvanted with type III (IFN-λ). Although mice showed mildly reduced clinical disease and decreased viral burden with PR8 immunization alone, those receiving PR8 with IFN-λ adjuvant showed no weight loss with primary challenge and nearly undetectable virus in upper and lower airway by day 5 postinfection (Supplemental Figure 13, G–J). These findings using a complementary approach suggest that the minimal IFN expression in the upper airway contributes to suboptimal immunization responses in 2 separate models of respiratory virus infection.

### Both T cell–mediated and humoral immunity contribute to IFN-driven improvements in mucosal vaccine responses.

**Figure 8:**
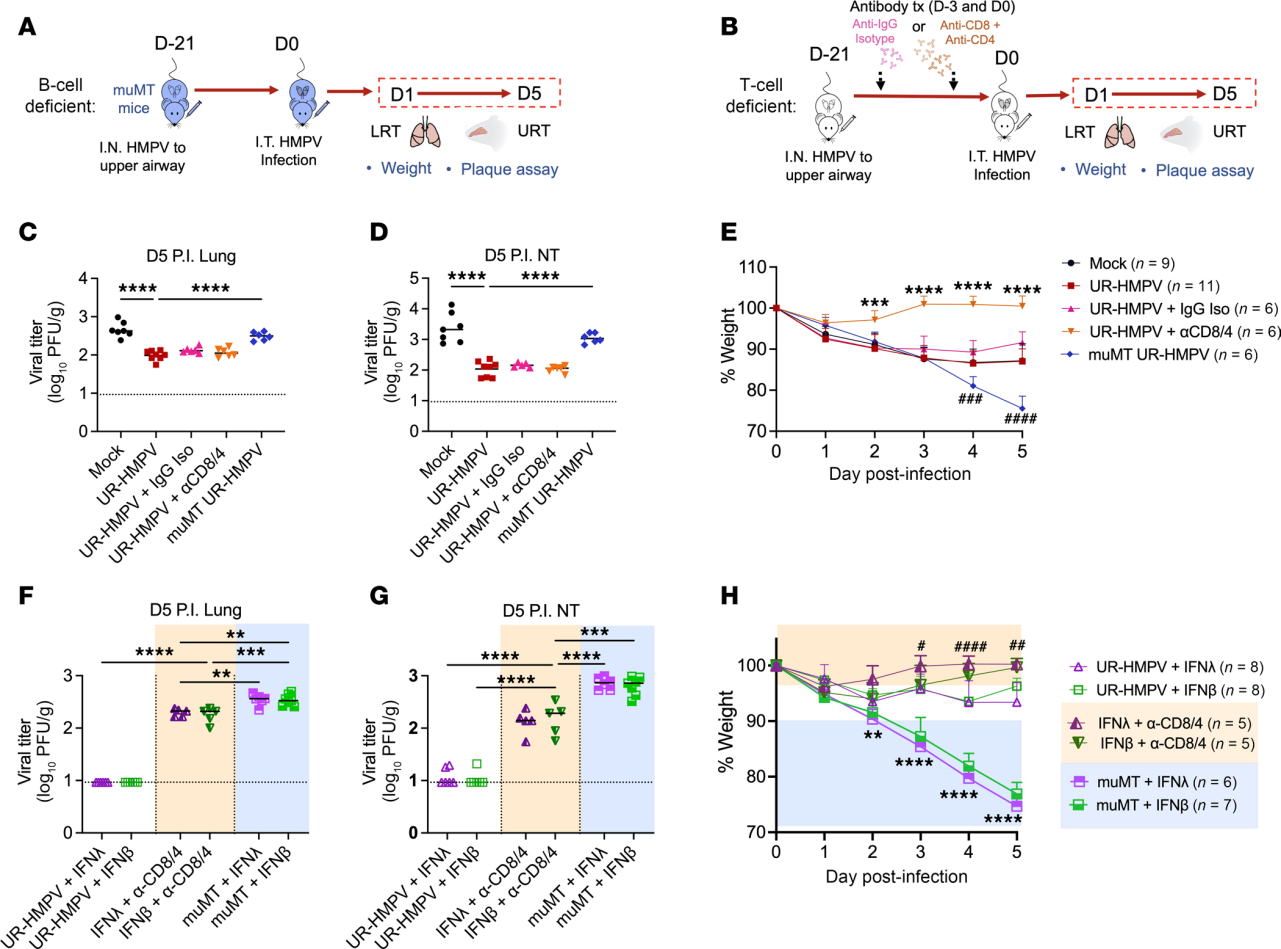
T cell–mediated and humoral immunity are both required for improved nasal HMPV vaccine response with IFN adjuvant. Mice were immunized as described in Figure 6A with low-dose HMPV (UR-HMPV) in either a B cell–deficient model of muMt^-^ (muMT) mice (**A**) or a T cell–deficient model with neutralizing CD4^+^ and CD8^+^ antibody depletion (**B**). IgG isotype control antibody was used as a negative control. HMPV titer (PFU/g) was measured in lungs (**C**) and nasal turbinates (**D**) of immunized mice day 5 postinfection. Limit of detection noted by dashed black line. (**E**) Disease was assessed by measuring body weight to day 5 postinfection, represented as % of day 0. ***P < 0.001, ****P < 0.0001 for T cell–deficient immunized mice versus other groups. ^###^*P* < 0.001, ^####^*P* < 0.0001 for B cell–deficient immunized mice versus other groups. (**F**–**H**) Mice received HMPV immunization adjuvanted with IFN-λ or IFN-β as previously described, in a T cell–deficient model (orange), or in a B cell–deficient model (blue). HMPV titer (PFU/g) was measured in lungs (**F**) and nasal turbinates (**G**) of IFN-adjuvanted immunized mice day 5 postinfection. Limit of detection noted by dashed black line. (**H**) Disease was assessed by measuring body weight to day 5 postinfection, represented as % of day 0. **P < 0.01, ****P < 0.0001 for B cell–deficient groups versus other groups. ^#^*P* < 0.05, ^##^*P* < 0.01, ^####^*P* < 0.0001 for IFN-λ–adjuvanted T cell–deficient mice versus other IFN-adjuvanted immunized mice groups. Analyses by 1-way or 2-way ANOVA. **P < 0.01, ***P < 0.001, ****P < 0.0001.

We sought to identify which aspect of adaptive immunity is critical for this IFN-driven augmentation of vaccine responses using HMPV infection and immunization of muMt^–^ mice, which lack mature B cells (Figure 8A). We and others have also shown that both CD4^+^ and CD8^+^ T cells contribute to HMPV clearance (20, 44). Thus, in parallel, we developed a model of T cell deficiency by antibody depletion of CD8^+^ and CD4^+^ T cells prior to primary challenge, confirming T cell depletion by flow cytometry (Figure 8B and Supplemental Figure 14). B cell–deficient HMPV-vaccinated mice, but not T cell–deficient mice, exhibited increased lung and nasal titers (Figure 8, C and D). However, T cell–deficient HMPV-vaccinated mice showed complete abolishment of clinical disease, while B cell–deficient mice (with intact T cell responses) exhibited worsened clinical disease (Figure 8E). Thus, both T and B cells contributed to adaptive responses to HMPV but in different capacities, with B cells being important to reduce viral burden and T cells promoting inflammatory antiviral responses that also contribute to pathology.

We next delivered IFN-λ– and IFN-β–adjuvanted vaccine in T cell– or B cell–deficient models and assessed HMPV burden. HMPV clearance from lungs and nasal airways by day 5 postinfection with IFN-adjuvanted vaccine delivery was abolished in both T and B cell–deficient mice (Figure 8, F and G). This verifies that both T cell–mediated and humoral immunity are essential for the observed decrease in HMPV burden, and thus improved mucosal vaccine response, with IFN augmentation. When assessing clinical disease, similar phenotypes were observed of complete disease reduction in T cell–deficient mice and worsened disease in B cell–deficient mice (with intact T cell response), with minimal effect by IFN adjuvant (Figure 8H). We also tested whether CD4^+^ or CD8^+^ T cells were more important for mucosal vaccine responses using separate models of CD4^+^ or CD8^+^ T cell depletion. Loss of CD4^+^ T cells resulted in complete disease reduction with IFN-adjuvanted HMPV vaccine, while CD8^+^ T cell–depleted mice still showed mild disease (Supplemental Figure 15, A and B). Both CD8^+^ and CD4^+^ T cell depletion abolished HMPV clearance from upper and lower tracts by day 5 postinfection with IFN-adjuvanted vaccine delivery, though CD8^+^-depleted mice showed a greater increase in lung and nasal HMPV burden (Supplemental Figure 15, C and D). Collectively, these data show that IFN adjuvant bolstered intranasal vaccine efficacy and antiviral immunity by promoting both T cell–mediated and humoral responses.

## Discussion

Here, we uncover that minimal IFN production in upper airways during respiratory viral infection contributes to a quiescent nasal immune landscape with limited innate and adaptive responses, low expression of antiviral and inflammatory cytokines, and delayed clearance. We show that this quiescence leads to suboptimal mucosal vaccine responses and highlight type I and III IFN as promising adjuvants to improve intranasal vaccination efficacy.

First, we employed a physiologically relevant mouse model of HMPV disease using the C2-202 HMPV clinical isolate, a viral strain isolated directly from patients (14, 15). C2-202 infection of mice, particularly at the dose used in this paper, results in a mild and self-limiting disease that resolves in 10–14 days, similar to human infection (23). We show using this model similarly high HMPV titers in both upper and lower airways, in accordance with previous studies of HMPV replication kinetics in mice (15, 21). However, despite high HMPV burden in nasal airway, there was minimal production of type I, II, or III IFN and, concordantly, limited expression of ISG and inflammatory cytokines in the upper respiratory tract. This starkly differed from IFN dynamics in the lung, where we showed that lung type I and III IFN were drastically increased in early HMPV infection (14, 15). Notably, we report similar findings of low IFN expression in nasal airways compared with lungs of influenza virus PR8–infected mice. We found that PR8 preferentially infects lower airways and replicates less efficiently in upper airways, similar to other mouse models of PR8 infection (45). However, despite this limitation, we developed a whole-airway model with efficient replication in both upper and lower airways that demonstrated similar upper tract immune quiescence as seen with HMPV. Studies of nasopharyngeal aspirates from HMPV-infected children also showed type I and III IFN upregulation, although HMPV B subtypes induced both type I and III IFN, while HMPV A groups mainly elicited type I IFN (46). Thus, further exploration of nasal IFN responses in both experimental models and clinical studies is needed, including possible differences between HMPV strains.

IFN responses were reduced in the upper compared with lower airway in HMPV-infected mice as well as in patients with severe COVID-19. Notably, there were distinct differences in cell populations between upper and lower tracts, with the lower airway exhibiting increased abundance of macrophages, T cells, and NK cells compared with the upper airway in both HMPV-infected mice and SARS-CoV-2–infected humans. Interestingly, upper airways were enriched with B cells and plasma cells. B cells are known to be the most abundant cell type within nasal-associated lymphoid tissues (NALTs), mucosal structures that can be found spread within nasal turbinates, and upper respiratory infections are known to increase B cell frequency and enhance germinal center formation in nasal airways (47, 48). A recent study showed that nasal turbinates play a key role in recruitment and homing of NALT-derived IgA-secreting plasma cells (49). While the roles of plasma and B cells in the upper airway are not fully elucidated, another study highlighted the importance of these cells residing within nasal tissue for protection against both respiratory and neurotropic pathogens (50).

Furthermore, we saw enrichment of genes that negatively regulate IFN production, particularly type III IFN–producing and cGAS/STING signaling pathways. Another study found that high type I and III IFN levels in upper airway in patients were associated with mild disease, but high IFN levels in the lower respiratory tract in patients were associated with severe COVID-19 (36). Similarly, type I and type II IFNs were reduced in nasopharyngeal aspirates of patients with severe RSV bronchiolitis, although this was also correlated to lower nasal titers (35). Other studies support the finding of suppressed nasal immune responses, with upper airway transcriptional data of patients with COVID-19 showing attenuated activation of antiviral responses, including Toll-like receptor, interleukin, and chemokine signaling (51). Studies of nasal immune responses in infants with RSV showed low expression of antiviral immune mediators, including macrophage, monocyte, and T cell chemoattractants (28).

We found that HMPV may suppress IFN production in nasal airways by reducing expression of IRF3, which is critical for IFN induction in HMPV-infected cells (52–54). Importantly, this finding was not observed in lower airway of the same HMPV-infected mice, suggesting this phenotype is upper airway specific. Several HMPV viral proteins, including G, M2-2, and SH, subvert immune recognition by downregulating IFN responses (55, 56). However, this study identifies evidence for an upper airway–specific IFN suppression strategy by HMPV. Understanding how HMPV inhibits IRF3 production, including which viral genes may be involved and how this response is directed to the upper airways alone, requires further exploration.

Through priming of nasal airways with recombinant type I or III IFN to mimic lung, we were able to rescue this quiescent immune environment. Mice receiving nasal IFN treatment showed increased recruitment of both total and virus-specific nasal CD8^+^ T cells and enhanced nasal HMPV clearance. The ability of type I IFN to prime adaptive immune response by enhancing DC recruitment, antigen presentation, and T cell activation has been well characterized (57). Here, we show that type III IFN is less inflammatory than type I yet can similarly enhance adaptive immune responses and reduce nasal HMPV burden (15, 42, 43, 58). This provides clinical promise for IFN-λ therapy to improve T cell immunity and antiviral responses to respiratory infections.

To assess whether the observed quiescent nasal immune environment impacts mucosal vaccine efficacy, we developed a method of nasal immunization using low-dose HMPV inoculum either alone or adjuvanted with type I or III IFN. Intranasal immunization required IFN adjuvant to prevent clinical disease and clear HMPV from upper and lower airways. IFN adjuvant increased serum neutralizing antibody and recruitment of nasal CD4^+^ and CD8^+^ T_RM_. Upper airway T_RM_ limit influenza virus spread to lower airway and reduce disease severity; thus, vaccines that induce T_RM_ possess greater potential for intranasal immunization (59). We showed that HMPV vaccine with IFN adjuvant enhances adaptive responses by increasing recruitment of cDC1 and pDC to the upper airway. Clinical studies of influenza virus and RSV showed increased mobilization of conventional and plasmacytoid DCs to the nasal mucosa following infection (60), suggesting nasal DCs are important for clearing virus from the upper airway. However, increased nasal DCs have been associated with recurrent wheezing following RSV bronchiolitis, indicating they can drive excessive inflammatory responses (61). The role of DCs, T_RM_ subsets, and other immune populations in the nasal airway and their contributions to nasal vaccine responses requires further characterization.

Intranasal vaccines remain an attractive route of immunization for respiratory viruses, allowing for noninvasive and targeted delivery to the respiratory tract (62). However, intranasal immunization has historically demonstrated limited therapeutic efficacy (39). Here, we show a likely contributor to suboptimal vaccine responses is the presence of a quiescent nasal immune landscape characterized by suppressed IFN production and minimal induction of antiviral effectors. Given suboptimal efficacy with current nasal vaccines, adjuvants are essential for bolstering immune responses in the nasal mucosa (40). In this murine model, nasal HMPV vaccine adjuvanted with type I or III IFN prevented clinical disease, promoted viral clearance, and augmented nasal vaccine responses. Thus, IFN-λ is a promising adjuvant in development of nasal vaccines for respiratory viral infections.

## Methods

### Sex as a biological variable

Our study examined both male and female animals, with similar findings observed and reported for both sexes.

### Viruses

HMPV isolate C2-202 (subtype B1, high virulence) was obtained from a patient with ARI, grown in LLC-MK2 cells, sucrose-purified, and titered by plaque assay as described (63, 64). Viral burden in mouse tissues was similarly titrated. HMPV stocks used in animal studies had all undergone <10 passages in LLC-MK2 cells. HMPV stocks used for experiments underwent a maximum of 3 freeze/thaw cycles, which do not significantly change viral titer (65). UV-HMPV was prepared by placing C2-202 virus stock in a 12-well tissue culture plate and placing the plate in the Stratagene 1800 UV Stratalinker. An automatic cross-linking protocol (1,200 × 100 μJ) was run 5 times, with 1-minute intervals on ice between cross-links to prevent the sample from heating up. UV-inactivated virus was titrated using LLC-MK2 plaque assay, and we confirmed that no replicating virus was present. PR8 (influenza H1N1) was provided by John Alcorn and Radha Gopal (University of Pittsburgh, Pittsburgh, Pennsylvania, USA).

### Animals and cells

Age-matched 6- to 8-week-old C57BL/6J (B6) mice and muMt^–^ (B6.129S2-Ighm^tm1Cgn^/J; strain 002288) mice were purchased from The Jackson Laboratory. All animals were maintained in specific pathogen–free conditions in accordance with University of Pittsburgh Institutional Animal Care and Use Committee guidelines. Cell lines used for experiments included LLC-MK2 monkey kidney cells (ATCC CCL-7) and C10 nontransformed mouse type II alveolar epithelial cells (noncommercial; donated by John Alcorn). LLC-MK2 cells were authenticated by ATCC short tandem repeat profiling prior to use. C10 cells were authenticated by flow cytometry staining for cell-specific alveolar type II epithelium markers prior to use.

### Infection and treatment models

For whole-airway HMPV infection, mice were anesthetized by inhaled isoflurane (5% isoflurane in 100% O_2_, flow rate 2.5 L/min) and infected intratracheally with 5 × 10^5^ PFU of C2-202 HMPV or mock LLC-MK2 cell lysate in 100 μL volume. For whole-airway influenza infection, mice were similarly anesthetized and infected intranasally (I.N.) with 2,000 PFU of PR8 or phosphate-buffered saline (PBS) for mock infection in 50 μL volume.

For IFN priming of the upper airways, mice were treated with either recombinant mouse IFN-β (Sino Biologicals; catalog 50708) or recombinant mouse IFN-λ2 (Peprotech; catalog 250-33) reconstituted in 0.1% bovine serum albumin (BSA). For delivery limited to the upper respiratory tract, anesthetized mice were given a 10 μL volume I.N. (5 μL per nostril) 1 day postinfection (15). Mock treatment involved same-day administration of an equal volume I.N. of 0.1% BSA alone. Mouse IFN-λ2 and IFN-β treatment was optimized to a similar degree of ISG induction, as described in Supplemental Figure 8; 1 μg of IFN-λ2 and 1 μg of IFN-β were administered to the upper airway.

### Vaccination models

For HMPV vaccination of the upper airways, mice were anesthetized, and a low dose of 2 × 10^5^ PFU C2-202 HMPV was delivered in an upper respiratory tract–restricted fashion as described previously by I.N. administration of a 10 μL volume (15). Mice were given mock vaccination with LLC-MK2 lysate, HMPV alone, or HMPV inoculum adjuvanted with 0.5 μg of either recombinant mouse IFN-λ2 or IFN-β 21 days before primary infection. To provide a B cell–deficient model, muMt^–^ mice were used for vaccination. To provide a T cell–deficient model, mice were vaccinated 21 days prior to infection as described and treated with a combination of 150 μg αCD8 (Bio X Cell; catalog BE0061) + 150 μg αCD4 (Bio X Cell; catalog BE0003-1) neutralizing antibodies or 300 μg rat isotype control in sterile PBS. Neutralizing antibodies were delivered to mice by intraperitoneal injection in a 100 μL total volume both 3 days prior to primary challenge and on the day of primary challenge.

For influenza vaccination of the upper airways, mice were similarly anesthetized, and a low dose of 800 PFU PR8 was delivered in an upper respiratory tract–restricted fashion I.N. in a 10 μL volume. Mock vaccination with sterile PBS, PR8 inoculum alone, or PR8 inoculum adjuvanted with 0.5 μg recombinant mouse IFN-λ2 was delivered to mice 21 days before primary infection.

### Flow cytometry

#### Single-cell preparation.

Lungs and nasal turbinates were simultaneously collected from euthanized mice. We developed and optimized a protocol for isolation of immune cells from nasal turbinates based on published studies (66–68). Following collection, nasal turbinates were flushed twice with 1 mL R10 (RPMI 1640 media supplemented with 10% FBS, 2 mM glutamine, 50 μg/mL gentamicin, 2.5 μg/mL amphotericin B, and 50 μM β-mercaptoethanol). Nasal turbinates were minced and digested with 2 mg/mL collagenase (Sigma-Aldrich), 20 μg/mL DNase (Roche), and 2 U/mL dispase (Corning) for 60 minutes in a shaker at 37°C. Lung tissue was minced and digested with 2 mg/mL collagenase and 20 μg/mL DNase for 60 minutes at 37°C. Both lung and nasal turbinate preparations were passed through a 70 μM cell strainer (BD Biosciences) and incubated with ACK lysis buffer (Sigma-Aldrich) to remove red blood cells.

#### Antibody staining.

Lymphocytes were first stained with APC-labeled tetramer against HMPV epitope H2-K^b^/M_94–102_ (VALDEYSKL) (M94) or APC-labeled flu NP-366 tetramer as irrelevant control for 90 minutes at room temperature (RT). Cells were next stained with innate or adaptive cell markers (Supplemental Table 1) using the following protocol: LIVE-DEAD violet dye (Thermo Fisher Scientific) for 20 minutes at RT, surface antibodies for 45–60 minutes at 4°C (1 μL antibody/sample in BD Horizon Brilliant Stain buffer) (catalog 566349), fixation with FOXP3 fix/permeabilization buffer for 20–60 minutes at 4°C (Invitrogen; catalog 50-112-8857), intracellular markers for 30–60 minutes at 4°C (1 μL antibody/sample in FOXP3 permeabilization buffer), and wash with FACS buffer (1% FBS in PBS). Fluorescence minus one (FMO) controls were prepared for resident memory markers, all inhibitory receptors, and all intracellular markers.

#### Ex vivo peptide stimulation.

In parallel with tetramer staining, 1 × 10^6^ lymphocytes from the same mouse sample were restimulated in vitro using M94 peptide in the presence of anti-CD107a (clone 1D4B, BD Biosciences), brefeldin A, and monensin (BD Biosciences) for 5 hours as described (23). Stimulation with flu NP-366 peptide was used as an irrelevant control. Cells were then stained for surface antibodies followed by intracellular cytokine staining for IFN-γ expression as described above.

Cells were resuspended in 300 μL in FACS buffer + 100 μL Counting Beads (BioLegend), strained through a nylon 70 μM filter (BD Biosciences), and analyzed on an Aurora spectral cytometer (Cytek Biosciences). Unstained cells from each experiment were collected, fixed with 4% paraformaldehyde, and used for spectral unmixing to subtract cellular autofluorescence. Data were analyzed using FlowJo software (Tree Star). FMO controls were used for gating of myeloid and lymphocyte subsets, inhibitory receptors, and transcription factors.

### Luminex immunoassay

Multiplex Luminex-based immunoassay (ProcartaPlex, Thermo Fisher Scientific) of undiluted lung homogenate or nasal turbinate homogenate was performed according to manufacturer’s instructions.

### qPCR

RNA was extracted from 100 μL volume of lung or nasal turbinate homogenate using RNeasy kit (QIAGEN) according to the manufacturer’s instructions. Quantitative reverse transcription PCR (RT-qPCR) was performed by preparing 25 μL reaction containing 5 μL extracted RNA using AgPath-ID One-Step RT-PCR Reagents (Applied Biosystems). TaqMan primers and probes were used according to the manufacturer’s instructions (Applied Biosystems). Flu (PR8) detection was done using InfA primer and probe set of the CDC rRT-PCR Flu Panel according to the manufacturer’s instructions (69). Values were normalized to the housekeeping gene *Hprt* and to mice receiving mock infection with cell lysate using the 2^–ΔΔCt^ method.

### siRNA knockdown

C10 cells were plated for 60%–80% confluence at transfection. Transfection of cells was performed using Lipofectamine RNAiMAX reagent (Thermo Fisher Scientific) and either Irf3 Silencer Select siRNA (Thermo Fisher Scientific; catalog 4390771-s79434) or Silencer Select Negative Control 1 siRNA (Thermo Fisher Scientific; catalog 4390843) according to manufacturer instructions. Transfected cells were infected with HMPV C2-202 at MOI (number of infectious viral particles per host cell) of 1.

### Neutralizing antibody assay

HMPV neutralizing antibody titers were determined through plaque reduction neutralization test as previously described (63). Serum was collected upon euthanasia of mice through intracardiac bleed. Sera were heat-inactivated at 60°C for 30 minutes. Serial 3-fold dilutions of heat-inactivated sera were prepared and mixed 1:1 with a working stock of C2-202 HMPV to give a combined concentration of 1 PFU/μL in total volume. The serum/virus mixture was incubated for 60 minutes at RT, and 50 μL was plated in triplicate on confluent LLC-MK2 monolayers in 24-well plates to deliver 50 PFU/well. Culture plates were incubated for 60 minutes at RT for virus adsorption, overlaid with 0.75% methylcellulose in OptiMEM (Thermo Fisher Scientific) supplemented with 5 μg/mL trypsin, and incubated at 37°C for 4 days, and titers were determined by plaque assay.

### scRNA-Seq

Single-cell suspensions were obtained from mouse nasal turbinates as described above for flow cytometric analysis. Cells were washed 5 times with 10 mL of PBS+10% FBS and centrifuged at 500*g* for 5 minutes, followed by a final wash and centrifugation at 200*g* for 10 minutes. Dead cells were removed by magnetic separation using Annexin V Kit (STEMCELL Technologies). scRNA-Seq was performed as described previously (15). Briefly, the 10x Genomics 3’ CellPlex Kit was used, and cells were tagged by lipid-conjugated barcode oligonucleotides according to manufacturer’s instructions. Cells were passed through a 40 μM filter, and cell viability was determined using Cellometer 2000 (all showing >90% viability) prior to loading on a 10x Genomics Chromium instrument. Following established techniques using Chromium Single Cell 3′ Library V2 kit (10x Genomics), libraries were constructed, and RNA-Seq was performed on each sample, targeting 20,000 reads per cell. Sequencing outputs were processed with Cell Ranger (10x Genomics).

### Western blot analysis

Whole protein was extracted from lung and nasal tissue homogenates by resuspension in RIPA buffer containing 1× Halt Protease and Phosphatase Inhibitor (78440, Thermo Fisher Scientific) and subsequently sonicated. Clarified cell lysates were prepared in 1× Laemmli SDS-PAGE buffer and denatured at 100°C for 5 minutes prior to gel loading. A total of 30 μg protein was loaded per well for SDS-PAGE and transferred onto PVDF membrane by Trans-Blot Turbo Transfer System (Bio-Rad). Western blots were done using anti-IRF3 (Cell Signaling Technology, catalog 39659S) and anti-IRF7 (Cell Signaling Technology, catalog 4302S) antibodies at 1:1,000 dilution in TBS-Tween buffer containing 5% nonfat powdered milk. Anti-vinculin antibody (catalog 13901T, Cell Signaling Technology) at 1:3,000 dilution was used as a loading control. Proteins were detected by chemiluminescent reaction using Femto Maximum Sensitivity Substrate (catalog 34095, Thermo Fisher Scientific) and imaged using Bio-Rad ChemiDoc MP Imaging System and Image Lab software. Quantification was done by ImageJ software (NIH).

### Statistics

All experiments were conducted in triplicate and repeated at least twice. scRNA-Seq was performed in duplicate, and data were analyzed using the Seurat pipeline (v4.0) in R. Individual samples were demultiplexed by barcode oligonucleotides. Doublets identified by hashtag oligos and poor-quality droplets (if deficient number of genes was detected) were excluded from analysis. Gene expression was assessed using Wilcoxon rank sum with Bonferroni correction. All other data analysis was performed using Prism version 9.0 (GraphPad Software). Individual comparisons were done using 2-tailed Student’s *t* test. Multiple-group comparisons were done using either 1-way or 2-way ANOVA with Tukey’s test. Extra sum-of-squares *F* test was used to test whether IC_50_ values differed between groups for neutralization assays. Data are represented as mean ± SD. *P* values less than 0.05 were considered significant.

### Study approval

All animal work was conducted in accordance with and approved by the University of Pittsburgh Institutional Animal Care and Use Committee.

### Data availability

Data values are available in the Supporting Data Values XLS file. scRNA-Seq data used in this paper are publicly accessible through NCBI Gene Expression Omnibus accession numbers GSE261511 (lung samples) and GSE305077 (nasal turbinate samples).

## Author contributions

JS conceived the project, designed and performed experiments, analyzed data, and wrote the manuscript. OBP performed experiments and revised the manuscript. TE analyzed data and revised the manuscript. JL provided study materials and revised the manuscript. MJ provided study materials and revised the manuscript. JVW conceived and designed experiments, interpreted data, supervised the project, acquired project funding, and revised the manuscript.

## Funding support

This work is the result of NIH funding, in whole or in part, and is subject to the NIH Public Access Policy. Through acceptance of this federal funding, the NIH has been given a right to make the work publicly available in PubMed Central.

NIH R01 AI085062 and R21 AI180460 (to JVW).

NIH T32 AI138954 (to JS and OBP).

NIH F30 HL159915-01A1 (to OBP).

NIH K12 HD000850 (to TE).

The Henry L. Hillman Foundation (to JVW).

UPMC Children’s Hospital of Pittsburgh Research Advisory Council fellowship award (to JS).

Pediatric Infectious Diseases Society SUMMERS Fellowship (to JS).

ARCS Scholarship (to OBP).

## Supplementary Material

Supplemental data

Unedited blot and gel images

Supporting data values

## Version 1 09/16/2025

In-Press Preview

## Version 2 10/22/2025

Electronic publication
