# Supplementary material for: Limited nasal IFN production contributes to delayed respiratory virus clearance and suboptimal vaccine responses
Source: JCI Insight. 2025 Sep 16;10(20):e182836. doi: 10.1172/jci.insight.182836 (PMC12581673; doi:10.1172/jci.insight.182836)
Supplement: Supplemental data [file jciinsight-10-182836-s202.pdf]

SUPPLEMENTAL FIGURE LEGENDS

Figure S1. Type I, II, and III IFN protein levels in the nasal airways are low or undetectable out to day 7 post-infection. Mice were infected with $5x10^5$ PFU C2-202 HMPV. Protein expression levels (ng/mL) of IFN-β (A), IFN-γ (B), and IFN-λ2/3 (C) were measured by Luminex immunoassay in nasal turbinate homogenates collected at indicated time points post-infection. 3 mice were used per time point. Limit of detection noted by dashed black line. Analyses by one-way ANOVA.

Figure S2. Gating strategies for mouse nasal innate and adaptive immune populations. A) Flow cytometric gating strategy for mouse nasal innate cells. We assessed frequency of macrophage, monocyte, and DC populations. B) Flow cytometric gating strategy for mouse nasal adaptive cells. We assessed frequency of NK, B-cells, and T-cells.

Figure S3. HMPV-infected nasal airways do not show increased innate or adaptive immune cell recruitment. A-G) Mice were infected with $5x10^5$ PFU C2-202 HMPV (or mock-infected with LLC-MK2 cell lysate), and nasal innate immune cell recruitment was assessed day 1 post-infection by flow cytometry. 7 mock-infected and 9 HMPV-infected mice were used. A-C) Frequency of DC subsets, including cDC1 (A), cDC2 (B), and pDC (C). D-G) Frequency of macrophage and monocyte subsets, including M1 macrophages (D), M2 macrophages (E), inflammatory monocytes (F), and resident monocytes (G). H-L) Mice were infected with $5x10^5$ PFU C2-202 HMPV (or mock-infected with LLC-MK2 cell lysate), and nasal adaptive immune cell recruitment was assessed day 10 post-infection by flow cytometry. 6 mock-infected and 7 HMPV-infected mice were used. Frequency of B-cells (H), NK cells (I), and T-cells (J) with further stratification by $CD4^+$ (K) and $CD8^+$ lymphocytes (L).

Figure S4. Upper and lower airway of HMPV-infected mice show distinct immune cell populations. Single cell RNA sequencing (scRNA-seq) was performed on cells isolated from nasal turbinates or lungs of mice infected with $5x10^5$ PFU C2-202 HMPV (or mock-infected with LLC-MK2 cell lysate) and harvested day 1 post-infection. Two mice per group (2 mock-infected, 2 HMPV-infected) were used for the scRNA-seq experiment. A) 18 subpopulations of immune and epithelial cells were defined, with some present only in nasal samples, and are represented by UMAP. B) Differential enrichment of transcripts represented by heat map, with clustering to define transcriptional profiles of 18 cellular subpopulations. C) Relative abundance levels were shown for mock- vs. HMPV-infected nasal airway (left) and lung (right).

Figure S5. HMPV-infected lower airways do not downregulate IRF3. IRF3 and IRF7 protein levels were measured in lungs of mice infected with $5x10^5$ PFU C2-202 HMPV or mock-infected day 1 post-infection by Western blot (A). Data was normalized to vinculin. B) Fold-change in area-under-curve (AUC) measurements of normalized IRF3 and IRF7 expression in mock- versus HMPV-infected lungs. Samples from 4 HMPV-infected mice were used for this experiment. Analyses done by student's t-test. * *P*<0.05.

Figure S6. **IRF3 knockdown decreases type I and III IFN production in HMPV infection**. siRNA-mediated knockdown of *Irf3* (or negative control siRNA) was performed on C10 non-transformed type II alveolar epithelial cells and validated by qPCR (A). B-C) Levels of *Ifnb1* (B) and *Ifnl3* (C) were quantified in *Irf3* siRNA-treated uninfected C10 cells (IRF3 KD), control siRNA-treated C10 cells infected with HMPV (Ctrl + HMPV), and *Irf3* siRNA-treated uninfected C10 cells infected with HMPV. Cells were infected using C2-202 HMPV at MOI of 1. Data is normalized to the HPRT1 gene and non-transfected cells by the $2^{-\Delta\Delta Ct}$ method. Analyses done by student's t-test or one-way ANOVA. * *P*<0.05, ** *P*<0.01, **** *P*<0.0001.

Figure S7. IRF3 expression is downregulated early post-infection in upper airway and can be induced by type I or III IFN. Expression levels of *Irf3* and *Irf7* were measured in lungs (A) and nasal turbinates (B) of mice infected with $5x10^5$ PFU C2-202 HMPV at early time points of 3, 6, and 12 hours post-infection by qPCR. Data is normalized to the HPRT1 gene and mock-infected mice by the $2^{-\Delta\Delta Ct}$ method. 4 mice per group were used for this experiment. C) Expression levels of *Irf3* and *Irf7* were measured in lungs and nasal turbinates of mice receiving 50-µL intranasal treatment with 1 µg IFN-λ or IFN-β on day 1 post-treatment. Data is normalized to the HPRT1 gene and mock-treated mice by the $2^{-\Delta\Delta Ct}$ method. 3 mice per group were used for this experiment. Analyses by one-way ANOVA or student's t-test. * *P*<0.05, ** *P*<0.01, **** *P*<0.0001.

Figure S8. Type I or III IFN treatment of upper airways show minimal ISG induction. Expression of *Irf9* was measured by qPCR in nasal turbinates of mice receiving 10-µL intranasal treatment with recombinant IFN-λ or IFN-β at various concentrations. Data is normalized to the HPRT1 gene and mock-treated mice by the $2^{-\Delta\Delta Ct}$ method. 3 mice per group were used for this experiment. Analyses by two-way ANOVA.

Figure S9. Type I IFN treatment of upper airways increases HMPV-specific $CD8^+$ T-cell recruitment and enhances viral clearance. Mice were infected with $5x10^5$ PFU C2-202 HMPV and treated intranasally with recombinant mouse IFN-β (1µg in 10 µL) day 1 post-infection or mock-treated with same volume 0.1% BSA to mimic, as shown by schematic (A). Disease was assessed by measuring body weight (B) to day 10 post-infection, represented as % of Day 0. HMPV titer (PFU/g) was measured in nasal turbinates of mock- or IFN-β-treated mice day 7 (C) and 10 (D) post-infection. Limit of detection noted by dashed black line. D-G) C2-202-infected mice receiving either mock or IFN-β treatment were euthanized day 7 post-infection and immune responses assessed. D) Frequency of total nasal $CD8^+$ T cells (left), virus-

specific (M94$^+$) CD8$^+$ T cells (middle), and inhibitory receptor-expressing HMPV-specific CD8+ T cells (right). 4 mock-treated and 6 IFN-β-treated mice were used for studies with HMPV-specific tetramer. E) Frequency of nasal CD8$^+$ T cells expressing CD107a (right) or IFNγ (left) after 5-hour *ex vivo* stimulation with HMPV M94 peptide. 5 mock-treated and 6 IFN-β-treated mice were used for *ex vivo* stimulation studies. F) Frequency of total nasal CD4$^+$ T cells (left) and differential CD4$^+$ subsets (right) Th1 (% Tbet$^+$ of CD4), Th2 (% GATA3$^+$ of CD4), Th17 (% RORγT$^+$ of CD4), and Treg (% FoxP3$^+$ of CD4). G) Frequency of EOMES expression (of total CD4, total CD8, and total M94-specific CD8). Analyses by student's t-test or two-way ANOVA. * P<0.05, ** P<0.01, *** P<0.001, **** P<0.0001.

Figure S10. Type I and III IFN treatment of upper airway increased total and HMPV-specific nasal CD8$^+$ T-cells. Mice were infected with 5×10^5 PFU C2-202 HMPV and were treated intranasally with recombinant mouse IFN-λ or IFN-β day 1 post-infection as previously described. Nasal immune responses were assessed day 10 post-infection. A-C) Frequency of total nasal CD8$^+$ T cells (A), virus-specific (M94$^+$) CD8$^+$ T cells (B), and inhibitory receptor-expressing HMPV-specific CD8+ T cells (C). D-E) Frequency of total nasal CD4$^+$ T cells (D) and differential CD4$^+$ subsets (E) Th1 (% Tbet$^+$ of CD4), Th2 (% GATA3$^+$ of CD4), Th17 (% RORγT$^+$ of CD4), and Treg (% FoxP3$^+$ of CD4). F) Frequency of EOMES expression (of total CD4, of total CD8, and of total M94-specific CD8). 4 mock-treated, 3 IFN-λ-treated, and 4 IFN-β-treated mice were used for this experiment. Analyses by one-way or two-way ANOVA. ** P<0.01, *** P<0.001, **** P<0.0001.

Figure S11. IFN adjuvant of nasal HMPV immunization increases lung HMPV-specific CD8$^+$ and CD4$^+$ T_{RM} populations. A) Schematic of nasal immunization models. Mice were intranasally immunized with low-dose C2-202 HMPV (UR-HMPV), low-dose HMPV + adjuvant with recombinant IFN-λ (0.5μg), low-dose HMPV + adjuvant with recombinant IFN-β (0.5μg), or

received mock immunization with LLC-MK2 lysate. Mice were challenged with C2-202 HMPV infection 21 days post-inoculation, and lung responses to primary challenge were assessed. B) Frequency of total lung $CD4^+$ T cells. C-D) Frequency of total lung $CD8^+$ T cells (C) and virus-specific ($M94^+$) $CD8^+$ T cells (D). E-F) Frequency of lung $CD4^+$ (E) and $CD8^+$ (F) resident-memory (T_{RM}) cells. 5 mock-immunized, 6 HMPV-immunized, 6 IFN-λ-adjuvanted, and 6 IFN-β-adjuvanted mice were used for this experiment. Analyses by one-way ANOVA. * P<0.05, *** P<0.001.

Figure S12. IFN adjuvant improves response to vaccination with UV-inactivated HMPV by reducing clinical disease and HMPV burden. A-C) Mice were intranasally immunized with low-dose UV-inactivated C2-202 HMPV (UV-HMPV), UV-HMPV + adjuvant with IFN-λ (0.5μg), UV-HMPV + adjuvant with IFN-β (0.5μg), or mock-immunized with LLC-MK2 cell lysate. Mice were challenged with HMPV infection 21 days post-inoculation, and nasal responses to primary challenge were assessed. A) Disease was assessed by measuring body weight to day 5 post-infection, represented as % of Day 0. ** P<0.01, **** P<0.0001 for IFN-β- and IFN-λ-adjuvanted groups vs. UV-HMPV immunization alone. #### P<0.0001 for IFN-β- and IFN-λ-adjuvanted groups vs. mock immunization. HMPV titer (PFU/g) was measured in nasal turbinates (B) and lungs (C) of immunized mice day 5 post-infection. Limit of detection noted by dashed black line. 6 mice per group were used for this experiment. D-F) Mice were treated intranasally with IFN-λ (0.5μg), IFN-β (0.5μg), or mock-treated with 0.1% BSA and challenged with HMPV infection 21 days post-treatment. D)) Disease was assessed by measuring body weight to day 5 post-infection, represented as % of Day 0. HMPV titer (PFU/g) was measured in nasal turbinates (E) and lungs (F) of mice day 5 post-infection. Limit of detection noted by dashed black line. 3 mock-treated, 4 IFN-λ-treated, and 4 IFN-β-treated mice were used for this experiment.

Figure S13. Influenza virus-infected upper airway shows minimal IFN production and improved nasal vaccine responses with IFN adjuvant. Mice were infected with 2000 PFU influenza virus (PR8) or mock-infected with PBS. A) PR8 burden was measured by qPCR of lung and nasal turbinates collected day 5 post-infection. Disease was assessed by measuring body weight (B) and clinical scoring (C) to day 5 post-infection. Weight represented as % of Day 0. Clinical severity scores were measured by assigning 1 point out of 5 for each of the following: hunching, huddling, fur ruffling, rapid breathing, and lethargy. D-F) *Ifng* (D), *Ifnb1* (E) and *Ifnl3* (F) expression in lung and nasal turbinates of PR8-infected mice were measured by qPCR days 1 and 5 post-infection. 3 mock-infected and 4 HMPV-infected mice were used for infection studies. G-J) Mice were intranasally immunized with a 10μL volume of low-dose PR8 (UR-Flu), low-dose PR8 + adjuvant with recombinant IFN-λ (0.5μg), or mock-immunization with equal-volume PBS. Mice were challenged with PR8 infection 21 days post-inoculation. Disease was assessed by measuring body weight (G) and clinical scoring (H) to day 5 post-infection. † p<0.05, †† p<0.01, ††† p<0.001 for mock vs. UR-Flu groups. * p<0.05, ** p<0.01, *** p<0.001, **** p<0.0001 for mock vs. IFN-λ-adjuvanted immunized mice . # p<0.05, ### p<0.001, #### p<0.0001 for UR-Flu alone vs. IFN-λ-adjuvanted groups. PR8 burden was measured by qPCR of lung (I) and nasal turbinates (J) collected day 5 post-infection. qPCR data is normalized to the *Hprt1* gene and mock-infected mice by the $2^{-\Delta\Delta Ct}$ method. 4 mice per group were used for immunization studies. Analyses by one-way or two-way ANOVA. * *P*<0.05, ** *P*<0.01, *** *P*<0.001, **** *P*<0.0001.

Figure S14. Validation of $CD8^+$ and $CD4^+$ T-cell antibody depletion. A) Representative flow gating is shown for $CD8^+$ and $CD4^+$ T cells receiving combination αCD8/αCD4 antibodies or IgG isotype control. Frequency of total $CD8^+$ (B) or $CD4^+$ lymphocytes (C). 3 mice per group were used. Analyses by student's t-test. ** *P*<0.01, *** *P*<0.001.

Figure S15. $CD4^+$ T cells promote inflammatory responses while $CD8^+$ T cells restrict HMPV burden in nasal vaccination. Mice received low-dose HMPV immunization adjuvanted with IFN-λ as previously described. Prior to primary challenge with HMPV 21 days post-immunization, mice received either $CD4^+$ or $CD8^+$ antibody depletion as described in Figure 8B. Disease was assessed by measuring body weight (A) and clinical scoring (B) to day 5 post-infection. Weight represented as % of Day 0. Clinical severity scores were measured by assigning 1 point out of 5 for each of the following: hunching, huddling, fur ruffling, rapid breathing, and lethargy. * $P<0.05$, ** $P<0.01$, *** $P<0.001$ for $CD4^+$-depleted vs. no-depletion IFN-λ-adjuvanted groups. ## $P<0.01$, ### $P<0.001$ $CD4^+$-depleted vs. $CD8^+$-depleted IFN-λ-adjuvanted groups. HMPV titer (PFU/g) was measured in lungs (C) and nasal turbinates (D) of immunized mice day 5 post-infection. Limit of detection noted by dashed black line. Analyses by one-way or two-way ANOVA. * $P<0.05$, ** $P<0.01$, *** $P<0.001$, **** $P<0.0001$.

Figure S1

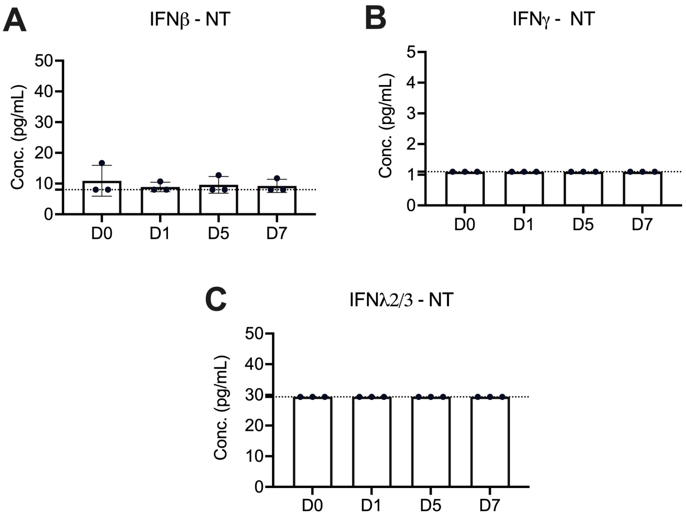
A
IFNβ - NT
Conc. (pg/mL)
50
40
30
20
10
0
D0
D1
D5
D7
B
IFNγ - NT
Conc. (pg/mL)
5
4
3
2
1
0
D0
D1
D5
D7
C
IFNλ2/3 - NT
Conc. (pg/mL)
50
40
30
20
10
0
D0
D1
D5
D7

Figure S2

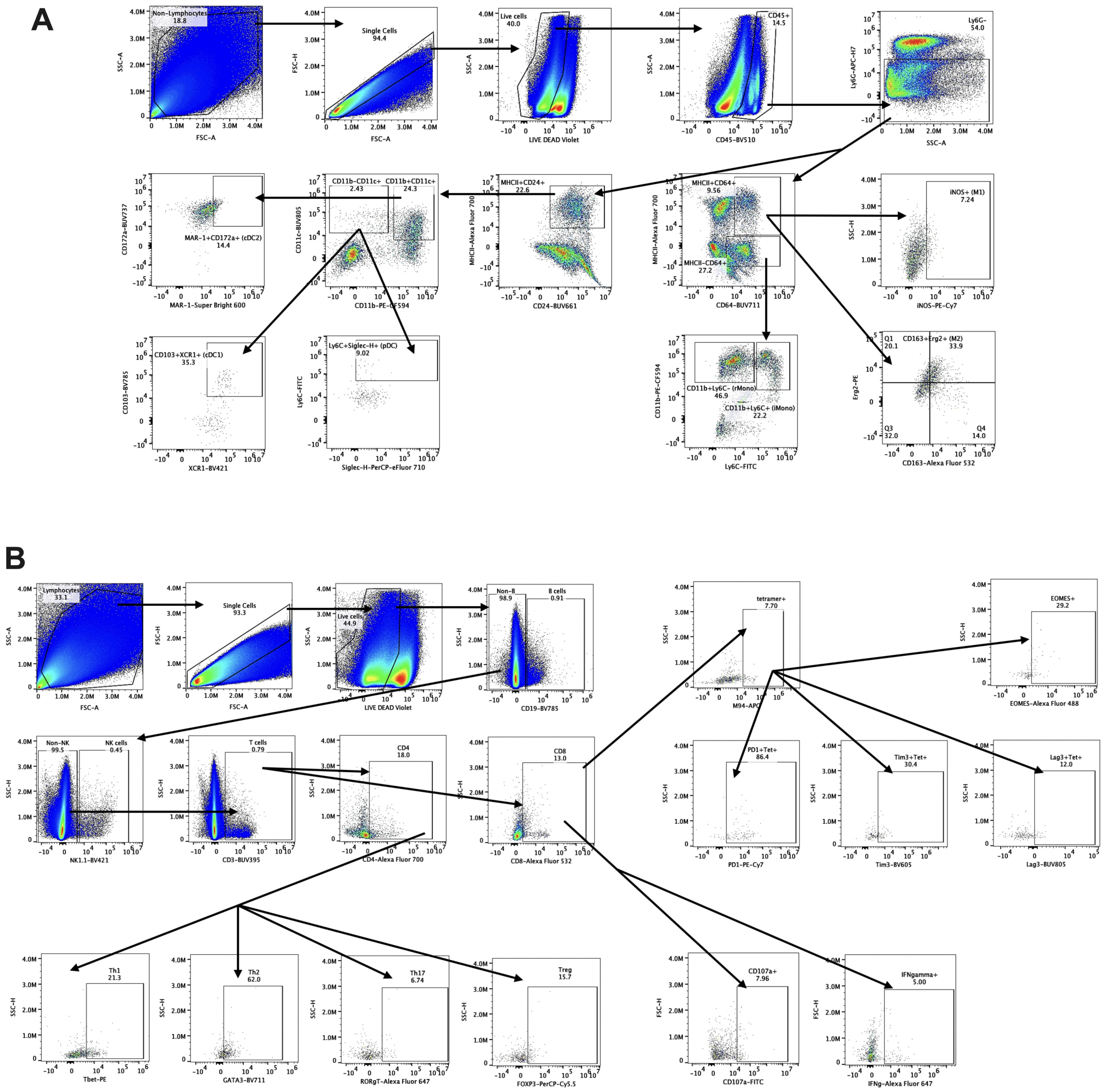
A
Non-Lymphocytes 18.8
Single Cells 94.4
Live cells 40.0
CD45+ 14.5
Ly6G- 54.0
FSC-A
SSC-A
FSC-H
LIVE DEAD Violet
CD45-BV510
Ly6G-APC-H7
MHCII+CD64+ 9.56
MHCII-CD64+ 27.2
CD64-BUV711
MHCII-Alexa Fluor 700
MHCII+CD24+ 22.6
CD24-BUV661
CD11b-CD11c+ 2.43
CD11b+CD11c+ 24.3
CD11b-PE-CF594
CD11c-BUV805
MAR-1+CD172a+ (cDC2) 14.4
MAR-1-Super Bright 600
CD172a-BUV737
iNOS+ (M1) 7.24
iNOS-PE-Cy7
SSC-H
CD103+XCR1+ (cDC1) 35.3
XCR1-BV421
CD103-BV785
Ly6C+Siglec-H+ (pDC) 9.02
Siglec-H-PerCP-eFluor 710
Ly6C-FITC
CD11b+Ly6C- (rMono) 46.9
CD11b+Ly6C+ (iMono) 22.2
CD11b-PE-CF594
Q1 20.1
CD163+Erg2+ (M2) 33.9
Q3 32.0
Q4 14.0
CD163-Alexa Fluor 532
Erg2-PE
B
Lymphocytes 33.1
Single Cells 93.3
Live cells 44.9
Non-B 98.9
B cells 0.91
CD19-BV785
Non-NK 99.5
NK cells 0.45
NK1.1-BV421
T cells 0.79
CD3-BUV395
CD4 18.0
CD4-Alexa Fluor 700
CD8 13.0
CD8-Alexa Fluor 532
tetramer+ 7.70
M94-APC
EOMES+ 29.2
EOMES-Alexa Fluor 488
PD1+Tet+ 86.4
PD1-PE-Cy7
Tim3+Tet+ 30.4
Tim3-BV605
Lag3+Tet+ 12.0
Lag3-BUV805
Th1 21.3
Tbet-PE
Th2 62.0
GATA3-BV711
Th17 6.74
RORgT-Alexa Fluor 647
Treg 15.7
FOXP3-PerCP-Cy5.5
CD107a+ 7.96
CD107a-FITC
FSC-H
IFNgamma+ 5.00
IFNg-Alexa Fluor 647

Figure S3

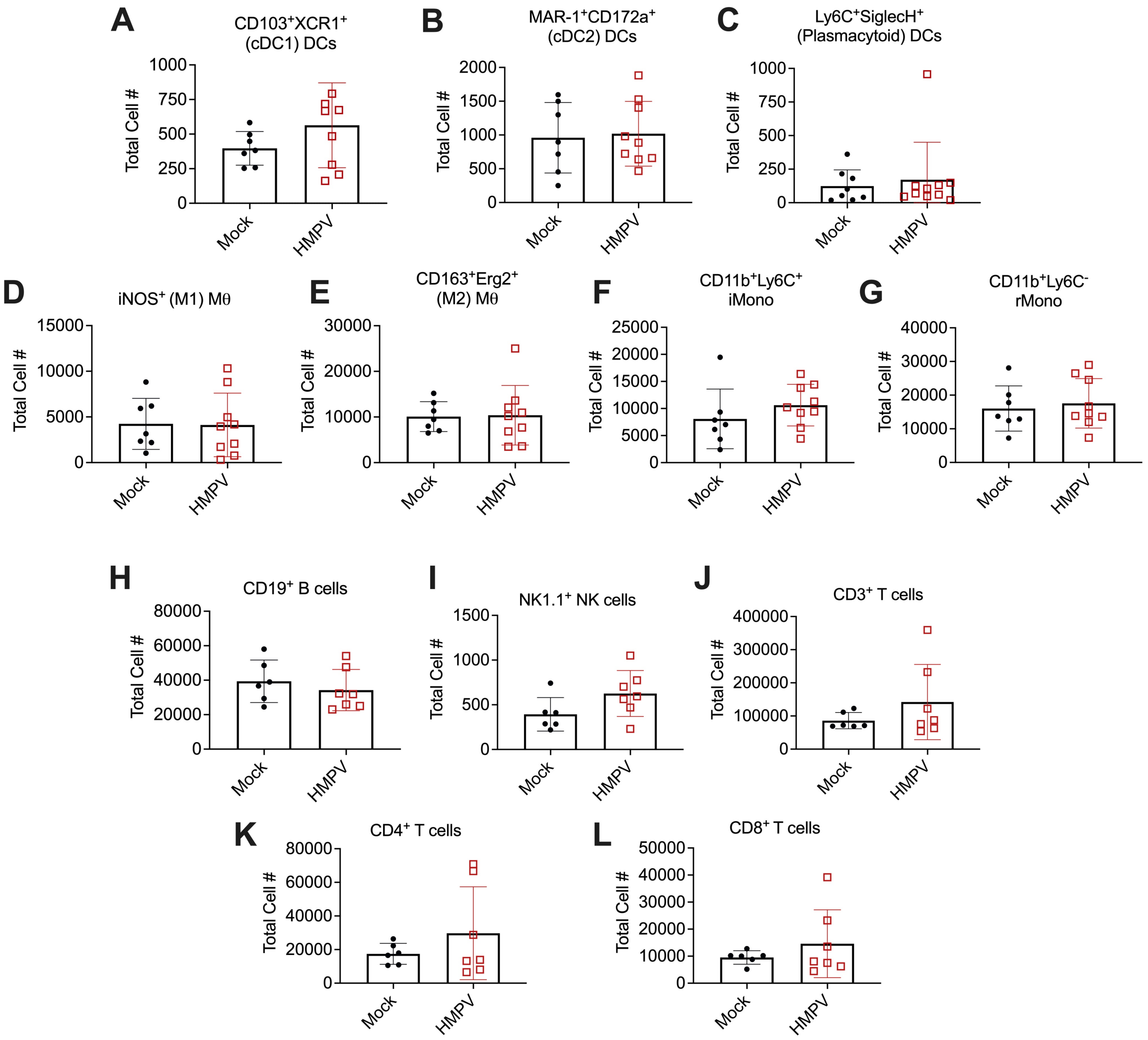

A
CD103+XCR1+ (cDC1) DCs
B
MAR-1+CD172a+ (cDC2) DCs
C
Ly6C+SiglecH+ (Plasmacytoid) DCs
D
iNOS+ (M1) Mθ
E
CD163+Erg2+ (M2) Mθ
F
CD11b+Ly6C+ iMono
G
CD11b+Ly6C- rMono
H
CD19+ B cells
I
NK1.1+ NK cells
J
CD3+ T cells
K
CD4+ T cells
L
CD8+ T cells
Total Cell #
Mock
HMPV

Figure S4

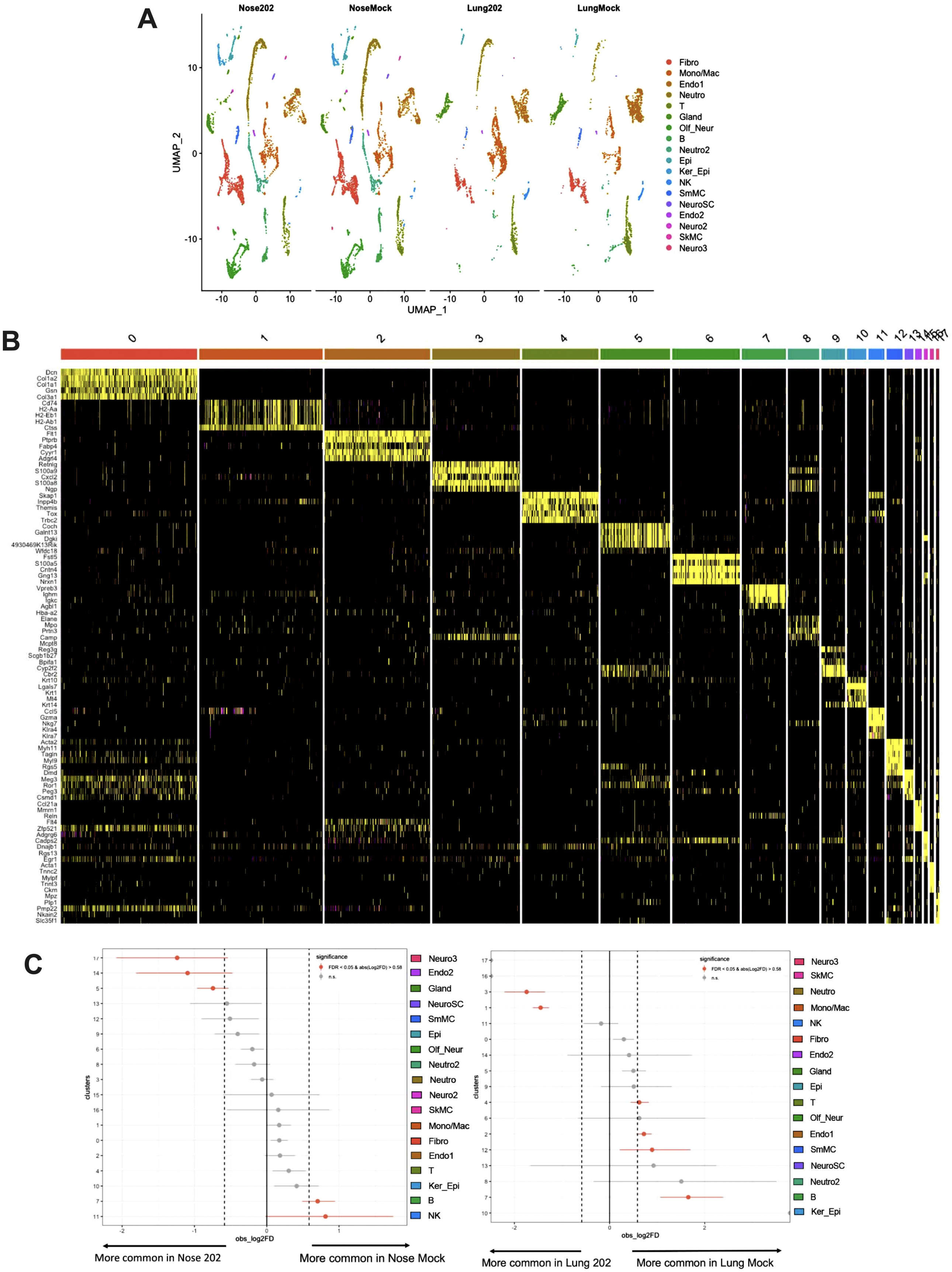
A
Nose202
NoseMock
Lung202
LungMock
UMAP_2
UMAP_1
Fibro
Mono/Mac
Endo1
Neutro
T
Gland
Olf_Neur
B
Neutro2
Epi
Ker_Epi
NK
SmMC
NeuroSC
Endo2
Neuro2
SkMC
Neuro3
B
C
significance
FDR < 0.05 & abs(Log2FD) > 0.58
n.s.
clusters
obs_log2FD
More common in Nose 202
More common in Nose Mock
More common in Lung 202
More common in Lung Mock

Figure S5

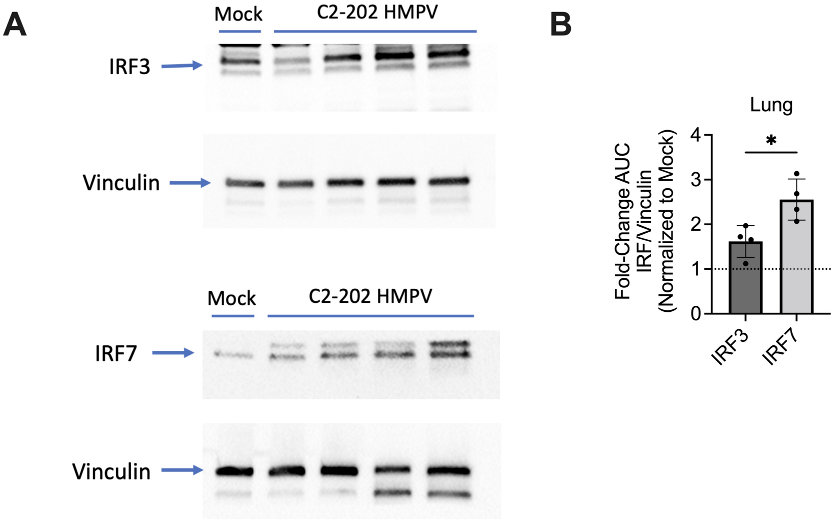
A
Mock
C2-202 HMPV
IRF3
Vinculin
Mock
C2-202 HMPV
IRF7
Vinculin
B
Lung
Fold-Change AUC
IRF/Vinculin
(Normalized to Mock)
*
0
1
2
3
4
IRF3
IRF7

Figure S6

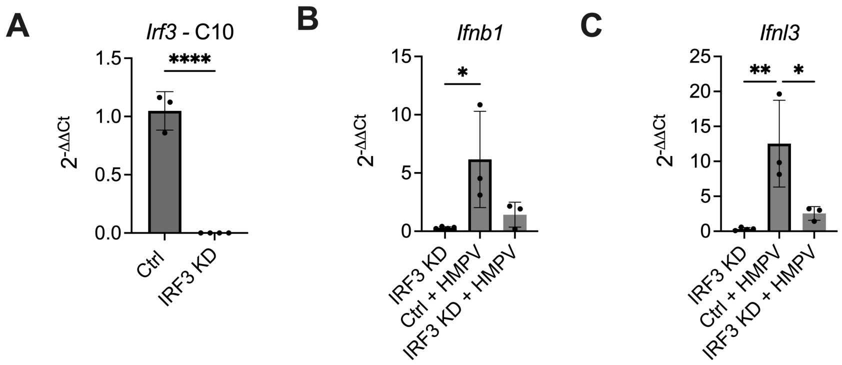
A
Irf3 - C10
****
1.5
1.0
0.5
0.0
$2^{-\Delta\Delta Ct}$
Ctrl
IRF3 KD
B
Ifnb1
*
15
10
5
0
$2^{-\Delta\Delta Ct}$
IRF3 KD
Ctrl + HMPV
IRF3 KD + HMPV
C
Ifnl3
**
*
25
20
15
10
5
0
$2^{-\Delta\Delta Ct}$
IRF3 KD
Ctrl + HMPV
IRF3 KD + HMPV

Figure S7

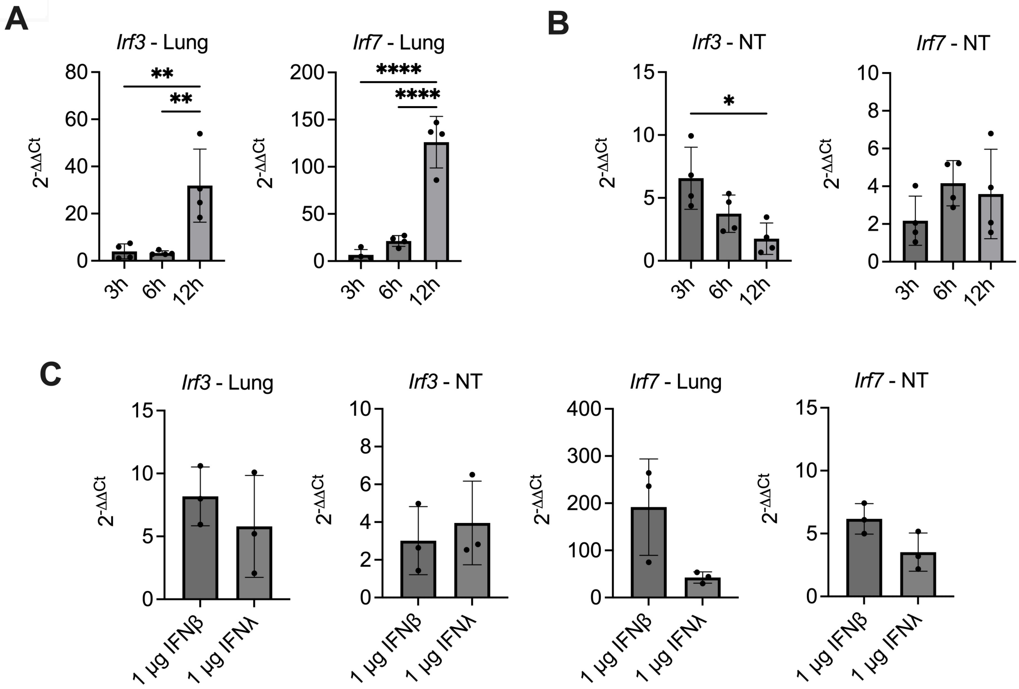
A
Irf3 - Lung
Irf7 - Lung
2^-ΔΔCt
3h
6h
12h
**
****
*
B
Irf3 - NT
Irf7 - NT
C
1 µg IFNβ
1 µg IFNλ

Figure S8

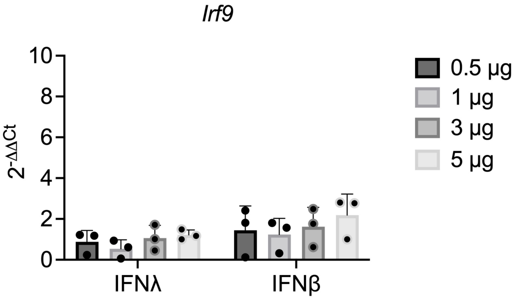

Figure S9

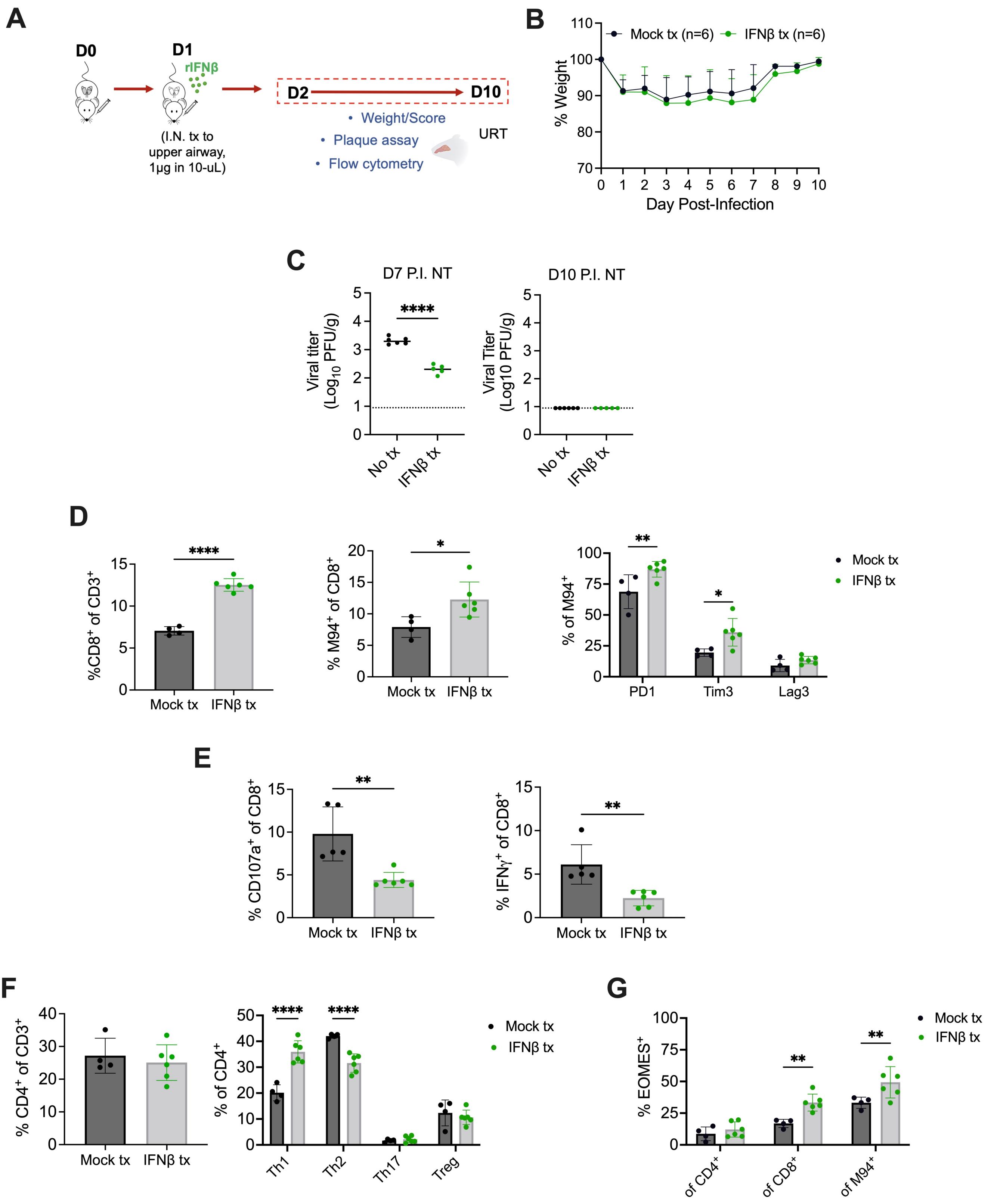

A
D0
D1
rIFNβ
(I.N. tx to
upper airway,
1μg in 10-uL)
D2
D10
• Weight/Score
• Plaque assay
• Flow cytometry
URT
B
Mock tx (n=6)
IFNβ tx (n=6)
% Weight
Day Post-Infection
C
D7 P.I. NT
D10 P.I. NT
Viral titer (Log10 PFU/g)
Viral Titer (Log10 PFU/g)
No tx
IFNβ tx
D
%CD8+ of CD3+
% M94+ of CD8+
% of M94+
Mock tx
IFNβ tx
PD1
Tim3
Lag3
E
% CD107a+ of CD8+
% IFNγ+ of CD8+
F
% CD4+ of CD3+
% of CD4+
Th1
Th2
Th17
Treg
G
% EOMES+
of CD4+
of CD8+
of M94+

Figure S10

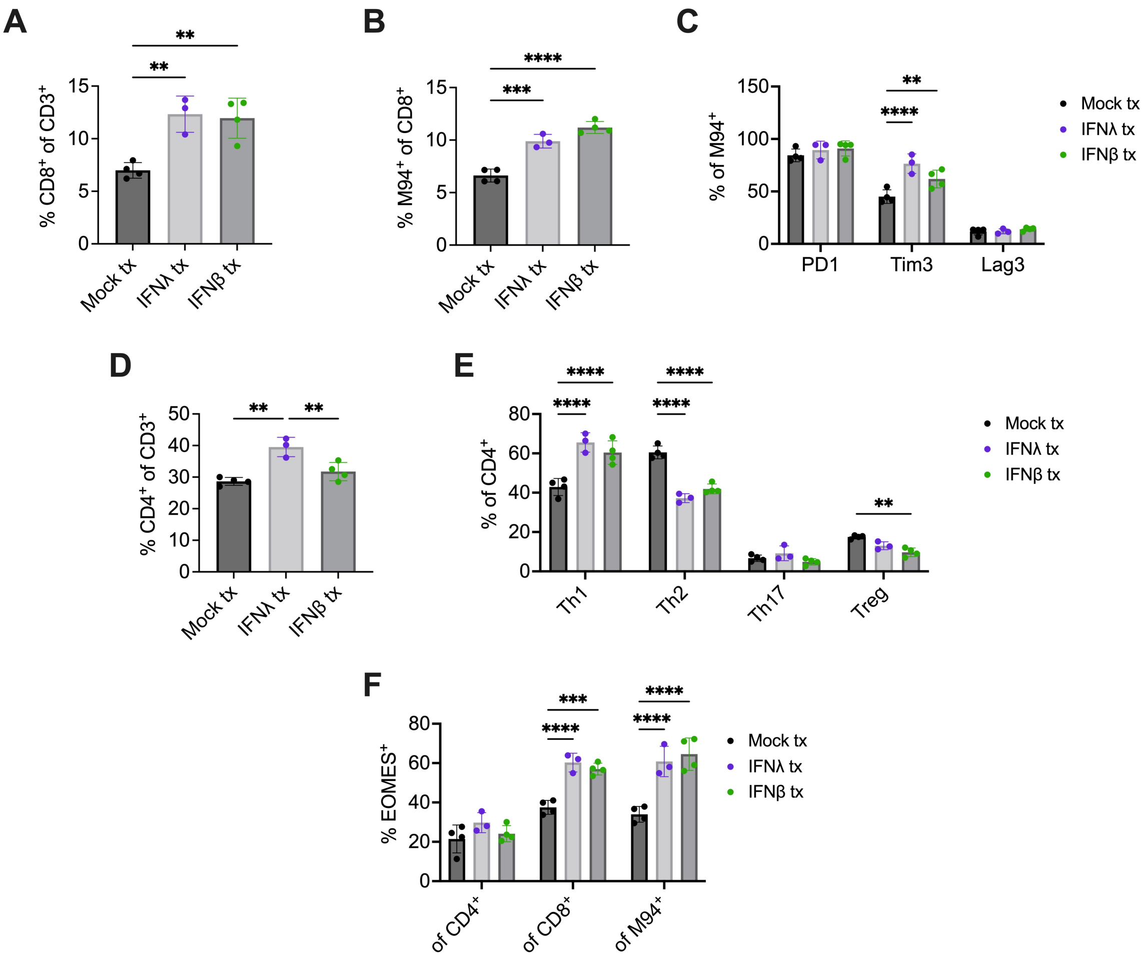
A
B
C
D
E
F
% CD8+ of CD3+
% M94+ of CD8+
% of M94+
% CD4+ of CD3+
% of CD4+
% EOMES+
Mock tx
IFNλ tx
IFNβ tx
PD1
Tim3
Lag3
Th1
Th2
Th17
Treg
of CD4+
of CD8+
of M94+
**
***
****

Figure S11

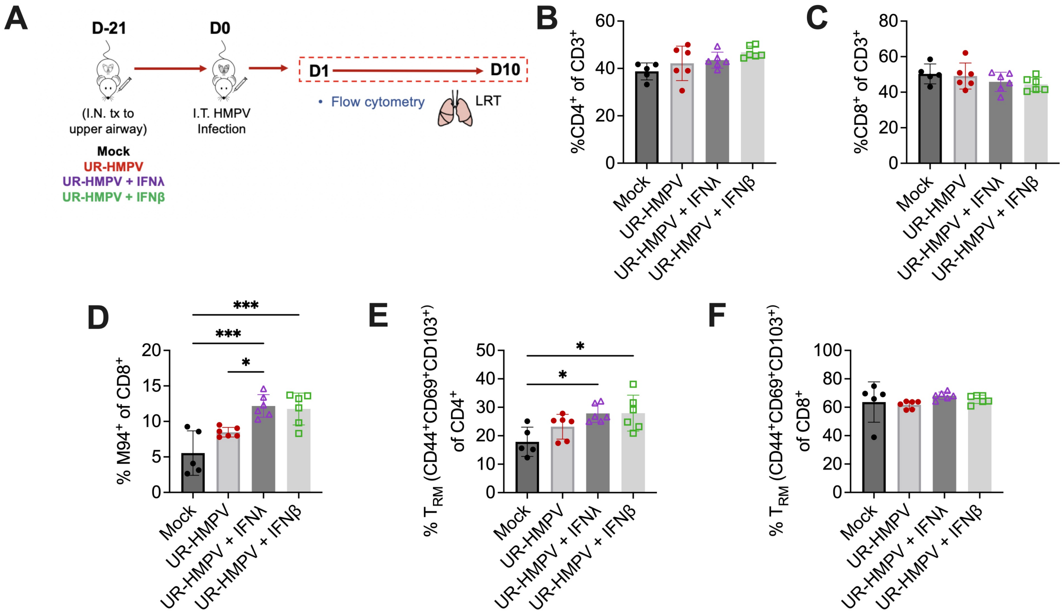

A
D-21
D0
D1
D10
(I.N. tx to upper airway)
I.T. HMPV Infection
• Flow cytometry
LRT
Mock
UR-HMPV
UR-HMPV + IFNλ
UR-HMPV + IFNβ
B
%CD4+ of CD3+
C
%CD8+ of CD3+
D
% M94+ of CD8+
***
***
*
E
% TRM (CD44+CD69+CD103+) of CD4+
*
*
F
% TRM (CD44+CD69+CD103+) of CD8+
Mock
UR-HMPV
UR-HMPV + IFNλ
UR-HMPV + IFNβ

Figure S12

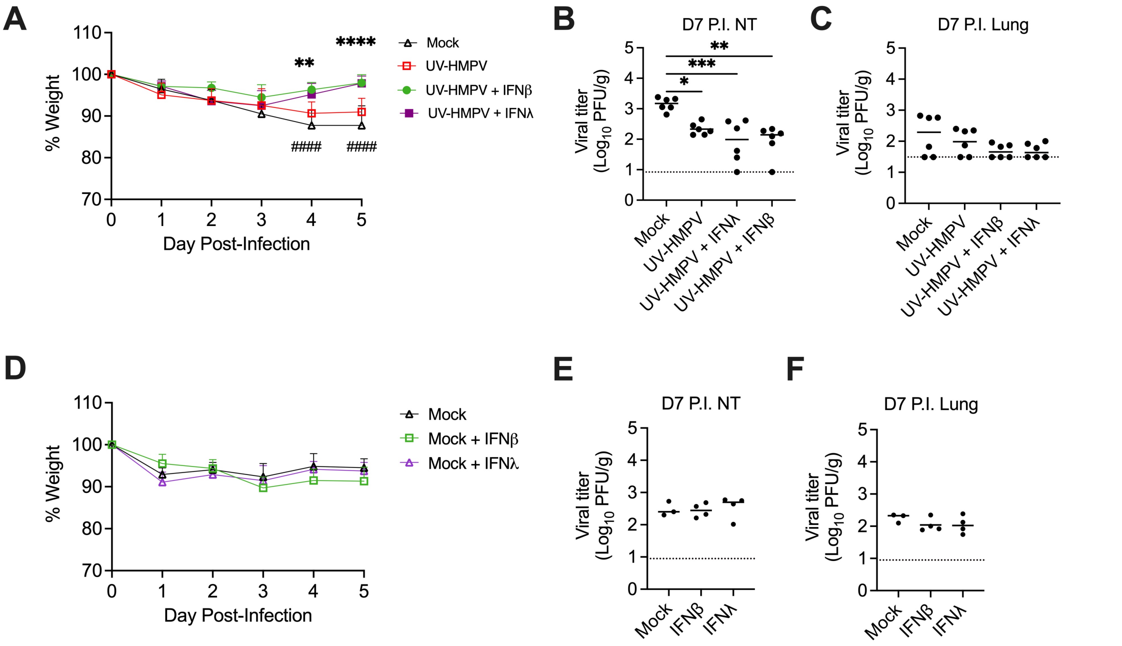
A
% Weight
110
100
90
80
70
0 1 2 3 4 5
Day Post-Infection
**
****
####
####
Mock
UV-HMPV
UV-HMPV + IFNβ
UV-HMPV + IFNλ
B
D7 P.I. NT
Viral titer (Log10 PFU/g)
*
***
**
Mock
UV-HMPV
UV-HMPV + IFNλ
UV-HMPV + IFNβ
C
D7 P.I. Lung
Viral titer (Log10 PFU/g)
Mock
UV-HMPV
UV-HMPV + IFNβ
UV-HMPV + IFNλ
D
% Weight
Day Post-Infection
Mock
Mock + IFNβ
Mock + IFNλ
E
D7 P.I. NT
Viral titer (Log10 PFU/g)
Mock
IFNβ
IFNλ
F
D7 P.I. Lung
Viral titer (Log10 PFU/g)
Mock
IFNβ
IFNλ

Figure S13

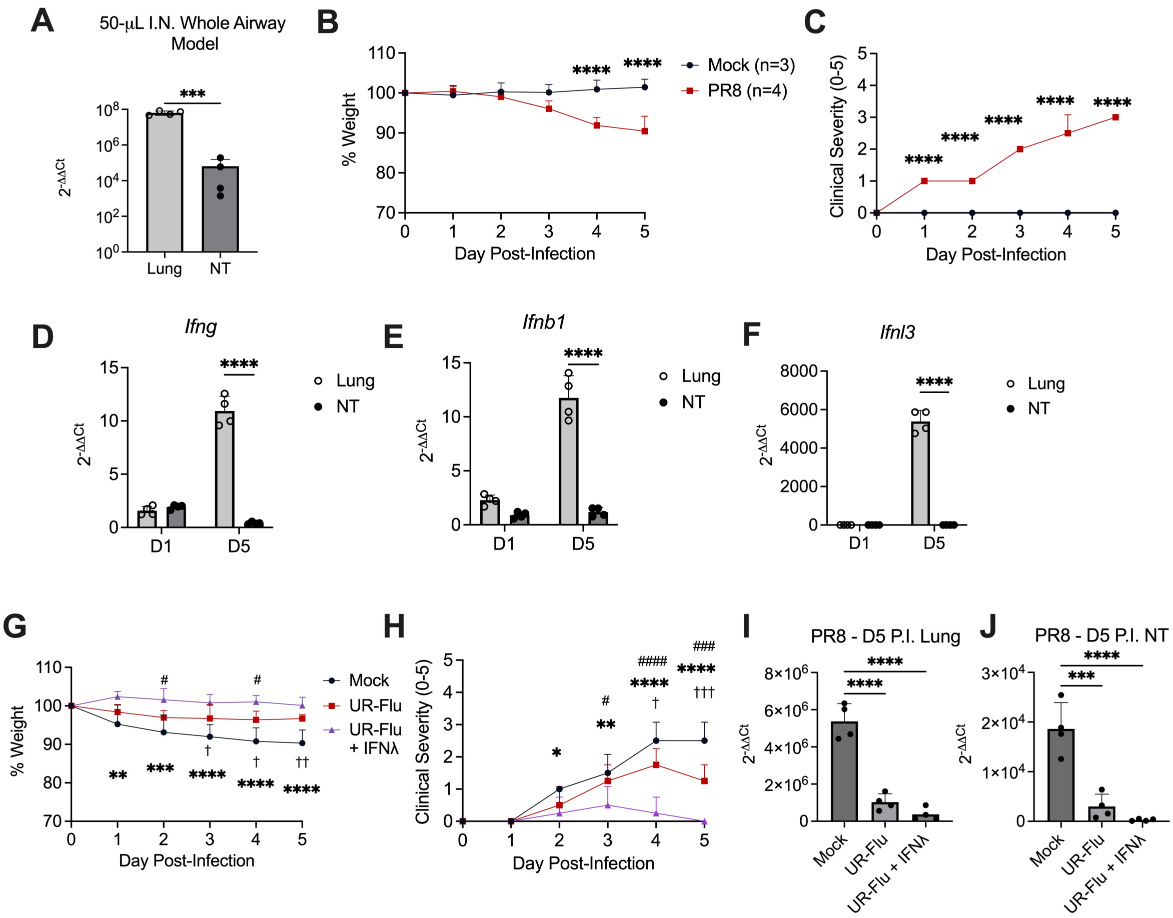

A
50-µL I.N. Whole Airway Model
Lung
NT
B
% Weight
Day Post-Infection
Mock (n=3)
PR8 (n=4)
C
Clinical Severity (0-5)
Day Post-Infection
D
Ifng
Lung
NT
D1
D5
E
Ifnb1
Lung
NT
F
Ifnl3
Lung
NT
G
% Weight
Day Post-Infection
Mock
UR-Flu
UR-Flu + IFNλ
H
Clinical Severity (0-5)
Day Post-Infection
I
PR8 - D5 P.I. Lung
Mock
UR-Flu
UR-Flu + IFNλ
J
PR8 - D5 P.I. NT
Mock
UR-Flu
UR-Flu + IFNλ

Figure S14

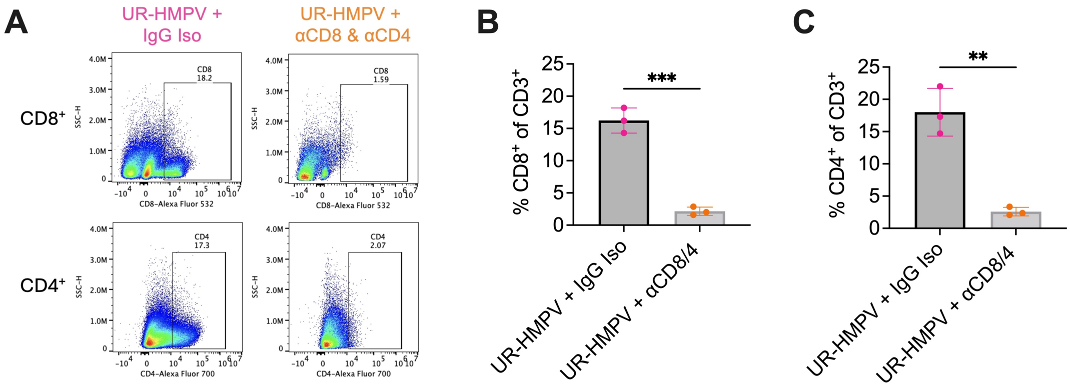

A
UR-HMPV + IgG Iso
UR-HMPV + αCD8 & αCD4
CD8⁺
CD4⁺
CD8 18.2
CD8 1.59
CD4 17.3
CD4 2.07
SSC-H
CD8-Alexa Fluor 532
CD4-Alexa Fluor 700
B
% CD8⁺ of CD3⁺
***
UR-HMPV + IgG Iso
UR-HMPV + αCD8/4
C
% CD4⁺ of CD3⁺
**
UR-HMPV + IgG Iso
UR-HMPV + αCD8/4

Figure S15

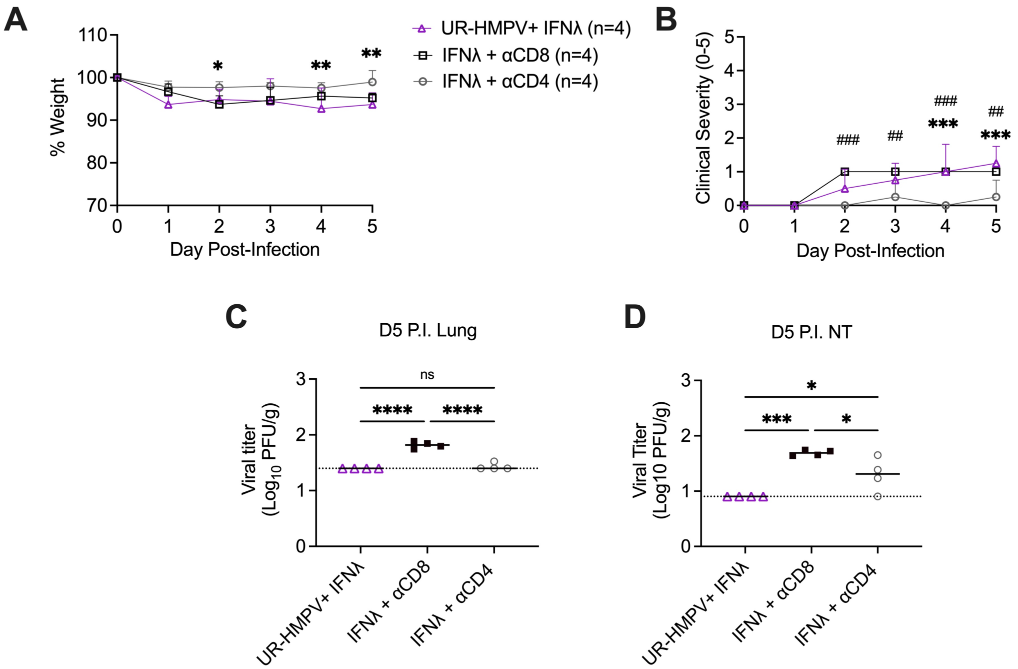
A
B
C
D
UR-HMPV+ IFNλ (n=4)
IFNλ + αCD8 (n=4)
IFNλ + αCD4 (n=4)
% Weight
Day Post-Infection
Clinical Severity (0-5)
D5 P.I. Lung
Viral titer (Log10 PFU/g)
D5 P.I. NT
Viral Titer (Log10 PFU/g)
ns
UR-HMPV+ IFNλ
IFNλ + αCD8
IFNλ + αCD4

Table S1. Antibody Table

Innate panel:					
Marker	Fluorophore	Species	Clone	Catalog #	Provider
CD45	BV510	Rat	30-F11	103138	BioLegend
Ly6G	APC-H7	Rat	1A8	565369	BD Biosciences
CD24	BUV661	Rat	MI/69	750679	BD Biosciences
MHCII	AF700	Rat	M5/114.15.2	107621	BioLegend
CD64	BV711	Mouse	X54-5/7.1	139311	BioLegend
CD11c	BUV805	Hamster	HL3	749090	BD Biosciences
CD11b	PE-CF594	Rat	MI/70	562287	BD Biosciences
iNOS	PE-Cy7	Rat	CXNFT	25-5920-82	Thermo-Fisher
CD163	AF532	Rat	TNKUPJ	58-1631-80	Thermo-Fisher
Erg2	PE	Rat	Erongr2	12-6691-80	Thermo-Fisher
MAR-1	Super Bright 600	Hamster	MAR-1	63-5898-82	Thermo-Fisher
CD172a	BUV737	Rat	P84	741819	BD Biosciences
Ly6C	FITC	Rat	AL-21	561085	BD Biosciences
Siglec-H	PerCP-eFluor710	Rat	eBio440c	46-0333-82	Thermo-Fisher
CD103	BV785	Hamster	2E7	121439	BioLegend
XCR1	BV421	Mouse	ZET	148216	BioLegend
Adaptive panel:					
CD19	BV785	Rat	6D5	115543	BioLegend
CD3	BUV395	Hamster	145-2C11	565992	BD Biosciences
CD8	AF532	Rat	53-6.7	58-0081-80	Invitrogen
CD4	AF700	Rat	RM4-5	100536	BioLegend
CD69	PE-Cy5	Hamster	H1.2F3	104510	BioLegend
CD44	APC-Cy7	Rat	IM7	560568	BD Biosciences
NK1.1	BV421	Mouse	PK136	562921	BD Biosciences
CD103	BUV661	Rat	M290	741504	BD Biosciences
PD1	PE-Cy7	Rat	RMP1-30	109110	BioLegend
Tim3	BV605	Mouse	5D12	747624	BD Biosciences
Lag3	BUV805	Rat	C9B7W	748540	BD Biosciences
FoxP3	PerCP-Cy5.5	Rat	FJK-16s	45-5773-80	eBioscience
Tbet	PE	Mouse	4B10	12-5825-80	eBioscience
GATA3	BV711	Mouse	L50-823	565449	BD Biosciences
RORgT	AF647	Mouse	Q31-378	562682	BD Biosciences
EOMES	AF488	Mouse	X4-83	567169	BD Biosciences
CD107a	FITC	Rat	1D4B	553793	BD Biosciences
IFNg	BV650	Rat	XMG1.2	505831	BioLegend
