# Supplementary material for: Limited nasal IFN production contributes to delayed respiratory virus clearance and suboptimal vaccine responses
Source: JCI Insight. 2025 Sep 16;10(20):e182836. doi: 10.1172/jci.insight.182836 (PMC12581673; doi:10.1172/jci.insight.182836)

Full unedited gel for Figure 4 – IRF3 Nasal Turbinates

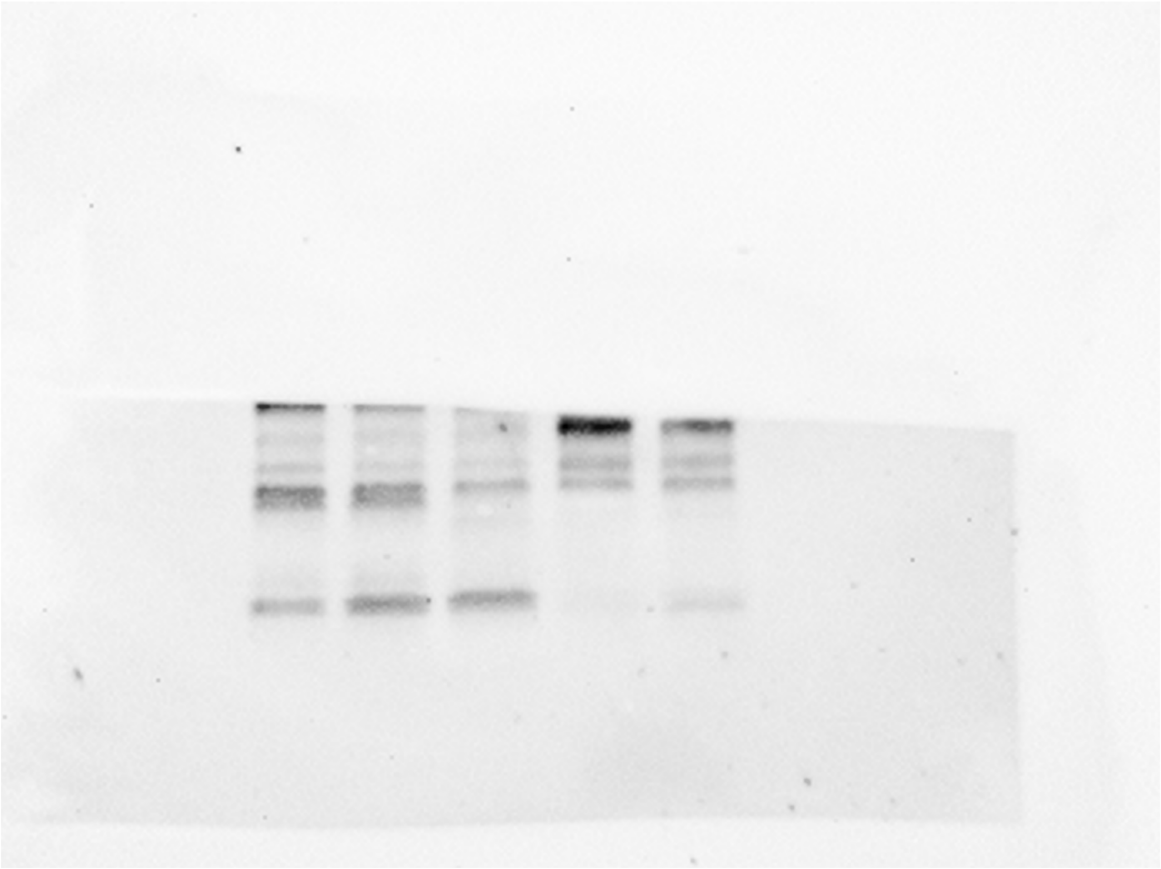

Full unedited gel for Figure 4 – vinculin (for IRF3 bands) Nasal Turbinates

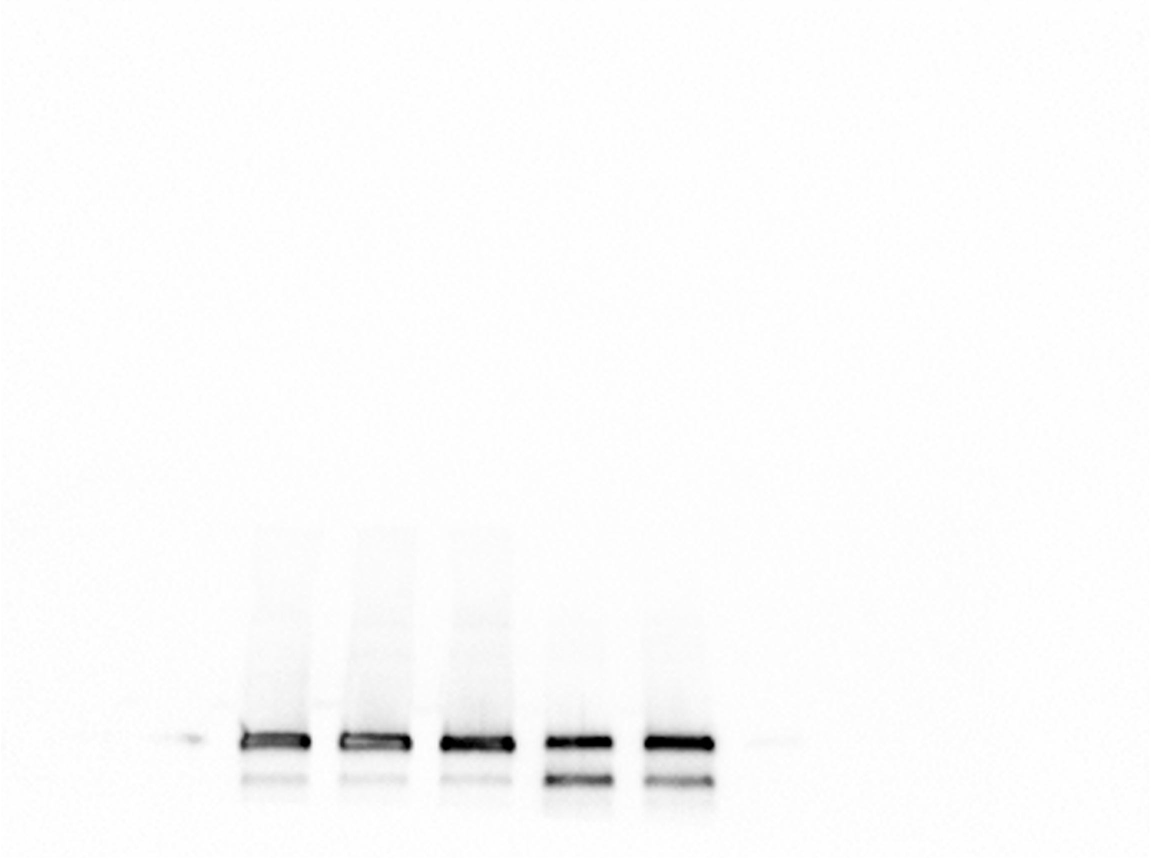

Full unedited gel for Figure 4 – IRF7 Nasal Turbinates

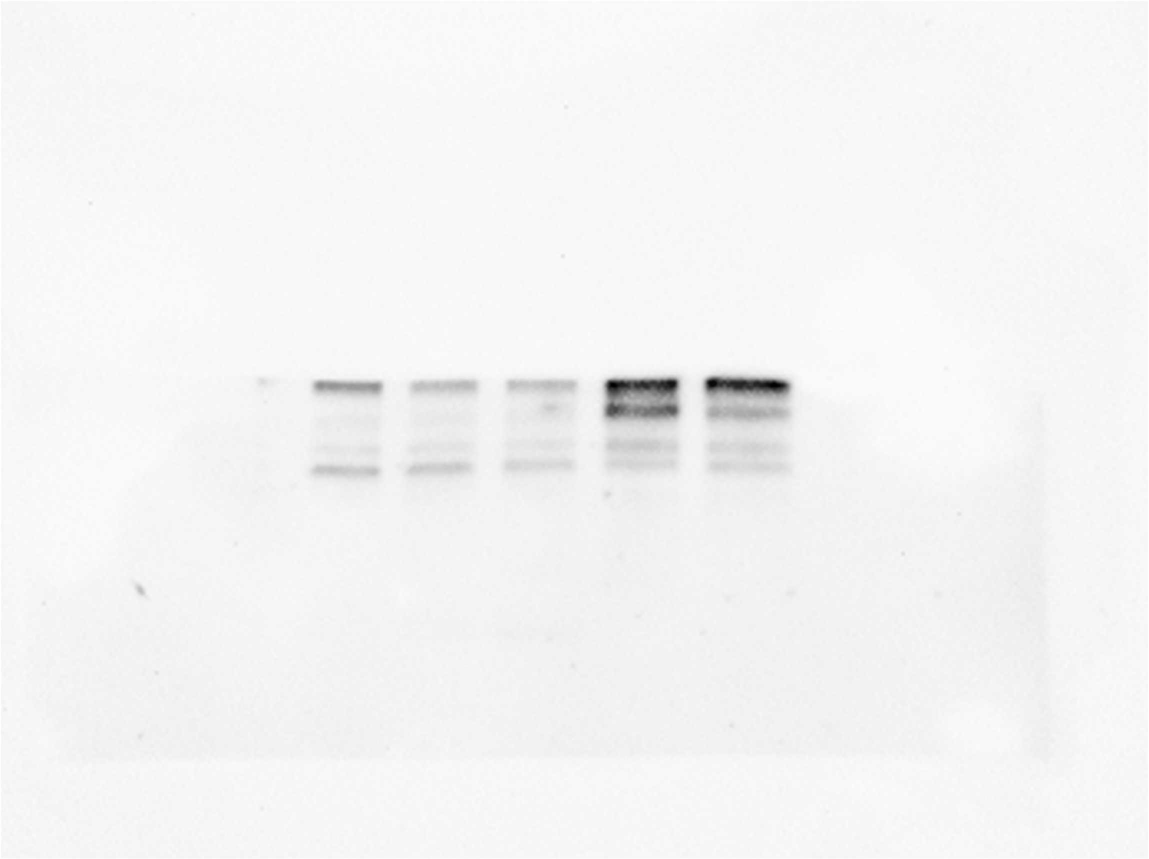

Full unedited gel for Figure 4 – vinculin (for IRF7 bands) Nasal Turbinates

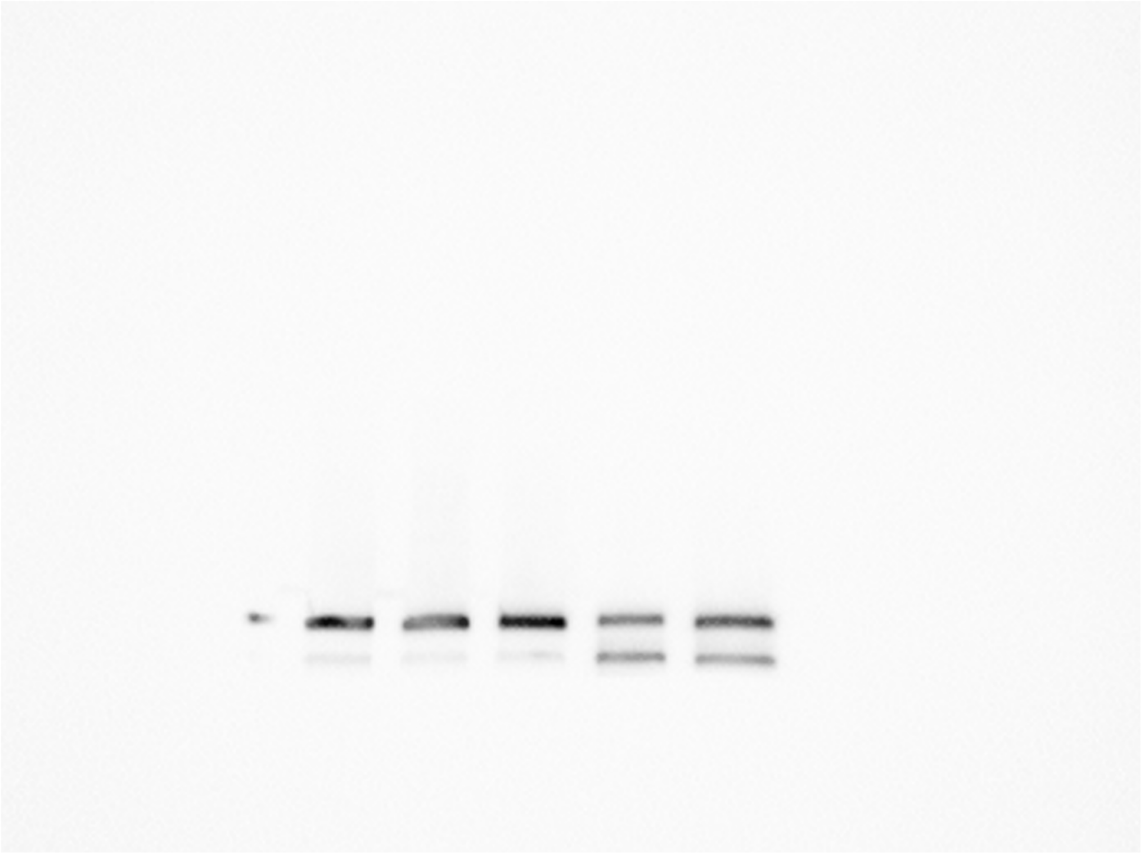

Full unedited gel for Figure S5 – IRF7 Lung

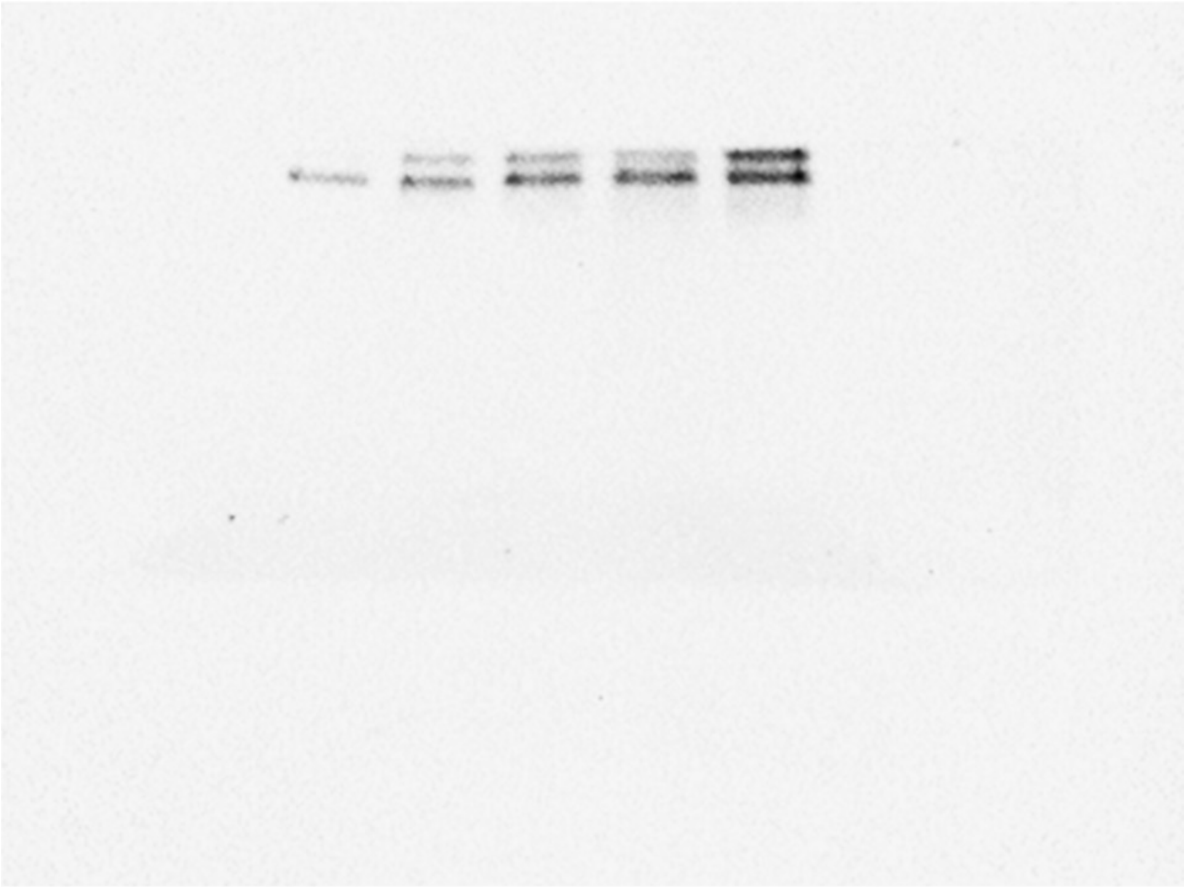

Full unedited gel for Figure S5 – vinculin (for IRF7 bands) Lung

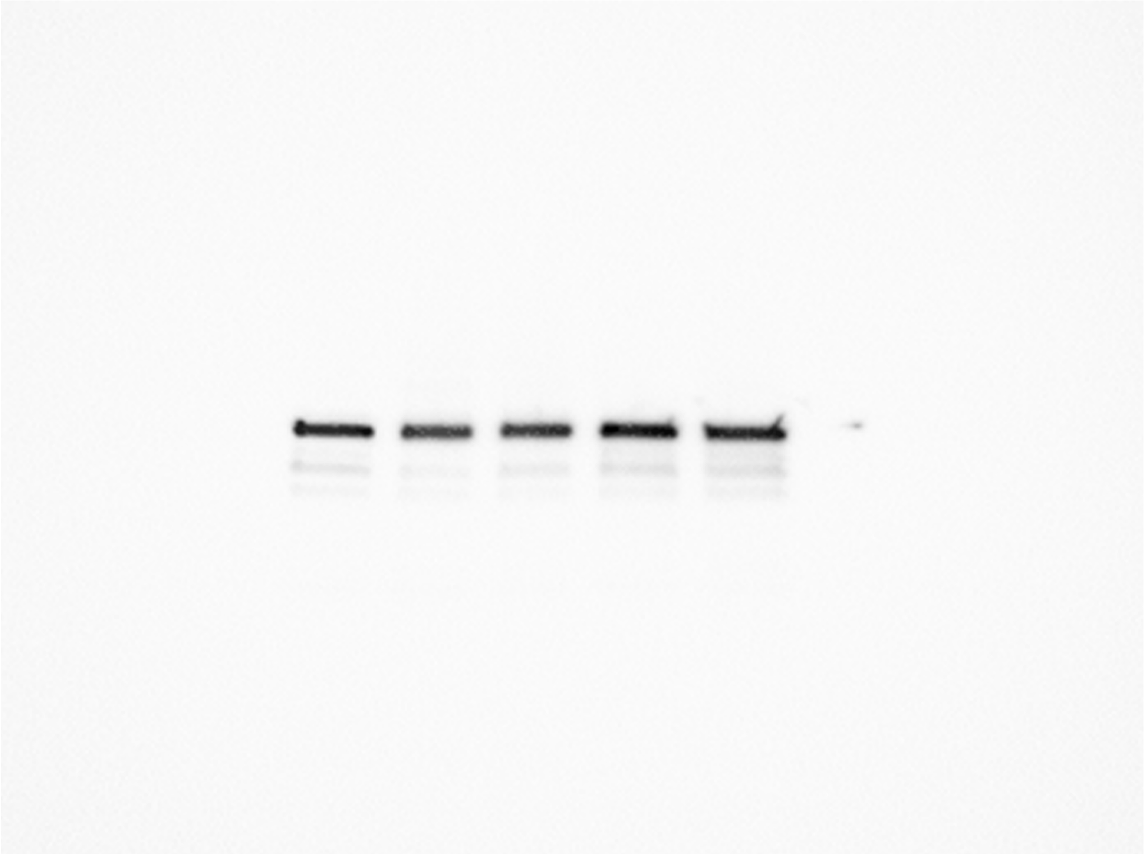

Full unedited gel for Figure S5 – IRF3 Lung

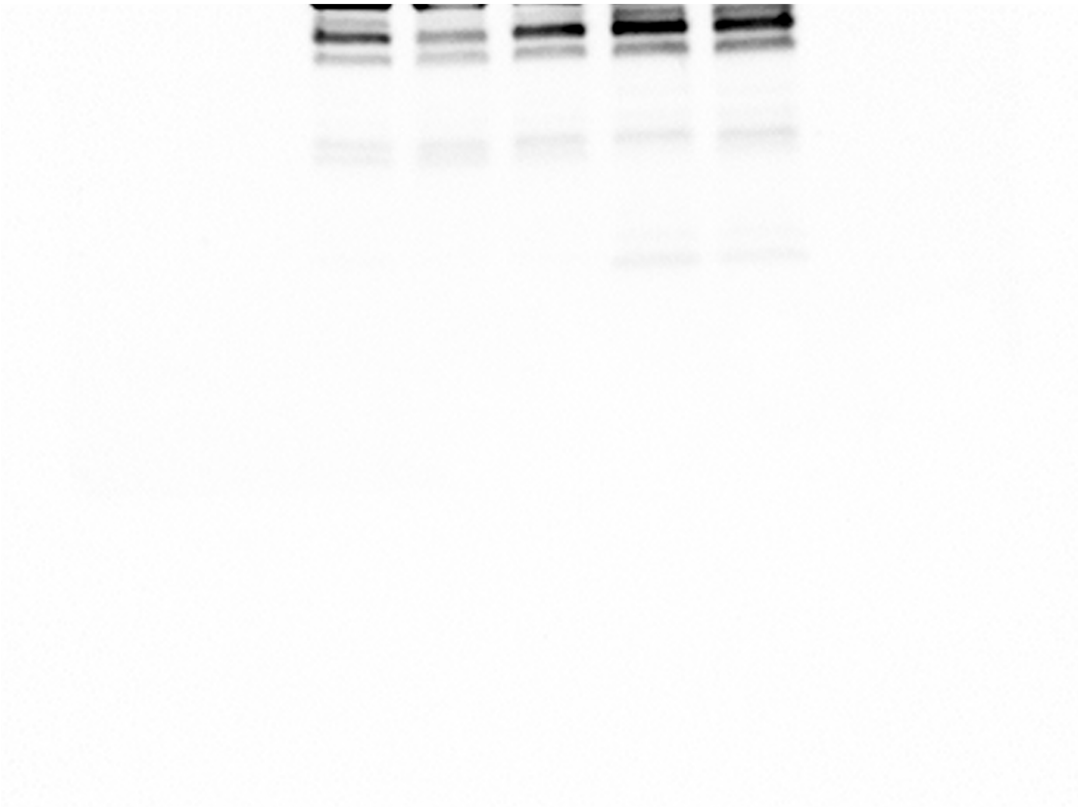

Full unedited gel for Figure S5 – vinculin (for IRF3 bands) Lung

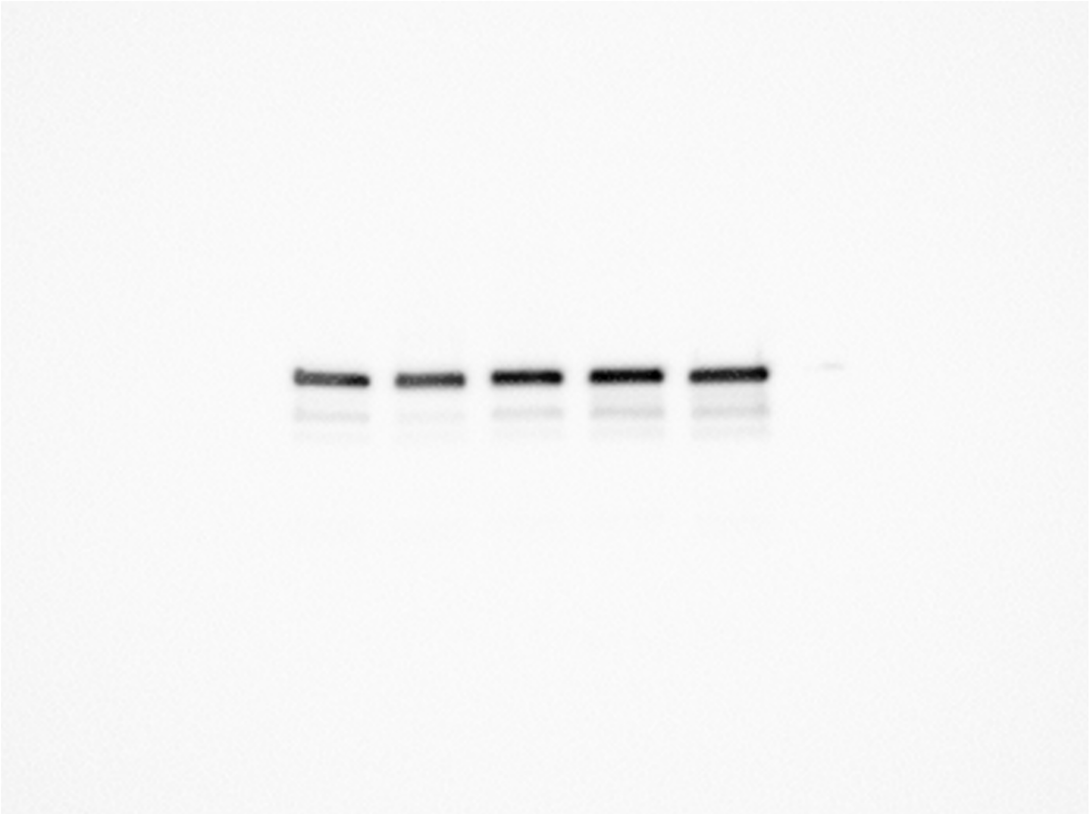

Supplement: Unedited blot and gel images [file jciinsight-10-182836-s203.pdf]
